# Prognostic factors of adherence to home-based exercise therapy in patients with chronic diseases: A systematic review and meta-analysis

**DOI:** 10.3389/fspor.2023.1035023

**Published:** 2023-03-24

**Authors:** Ellen Ricke, Arie Dijkstra, Eric W. Bakker

**Affiliations:** ^1^ Department of Social Psychology, University of Groningen, Groningen, Netherlands; ^2^ Department of Epidemiology and Data Science | Division EBM, Academic Medical Centre, Amsterdam, Netherlands

**Keywords:** chronic disease, exercise therapy, home-based, adherence, compliance, prognostic factor

## Abstract

**Background:**

Patients with a chronic disease may have an increased risk of non-adherence to prescribed home-based exercise therapy. We performed a systematic review with the aim to identify variables associated with adherence to home-based exercise therapy in patients with chronic diseases and to grade the quality of evidence for the association between these prognostic factors and adherence.

**Methods:**

Cohort studies, cross-sectional studies and the experimental arm of randomized trials were identified using a search strategy applied to PubMed, Embase, PsychINFO and CINAHL from inception until August 1, 2022. We included studies with participants ≥18 years with a chronic disease as an indication for home-based exercise therapy and providing data on prognostic factors of adherence to home-based exercise. To structure the data, we categorized the identified prognostic factors into the five WHO-domains; (1) Patient-related, (2) Social/economic, (3) Therapy-related, (4) Condition-related, and (5) Health system factors. Risk of bias was assessed using the Quality in Prognostic Studies (QUIPS) tool. Prognostic factors of adherence were identified and the quality of the evidence between the prognostic factors and adherence were graded using the Grading of Recommendations Assessment, Development, and Evaluation (GRADE) framework for predictor studies. We performed a meta-analysis of the obtained information.

**Results:**

A total of 57 studies were included. Within patient-related factors moderate- and high-quality evidence suggested that more self-efficacy, exercise history, motivation and perceived behavioral control predicted higher adherence. Within social-economic factors moderate-quality evidence suggested more education and physical health to be predictive of higher adherence and within condition-related factors moderate- and low-quality evidence suggested that less comorbidities, depression and fatigue predicted higher adherence. For the domains therapy-related and health-system factors there was not enough information to determine the quality evidence of the prognostic factors.

**Conclusion:**

These findings might aid the development of future home-based exercise programs as well as the identification of individuals who may require extra support to benefit from prescribed home-based exercise therapy.

**Systematic Review Registration:**

https://www.crd.york.ac.uk/prospero/display_record.php?RecordID=277003, identifier PROSPERO CRD42021277003.

## 1. Introduction

Chronic diseases represent the major share of burden of disease in Europe and are responsible for 86% of all deaths (1). There is accumulating evidence that in patients with chronic diseases exercise therapy is effective in improving the prognostic risk factor profile and, in certain diseases, in delaying mortality (2). According to the systematic review of Jolliffe et al. (3) exercise therapy in cases of documented coronary heart disease reduced all-cause mortality by 27% and total cardiac mortality by 31%. Exercise therapy is defined as systemic execution of planned physical movements, postures, or activities intended to enable the patient to reduce risk, enhance function, remediate or prevent impairment, optimize overall health, and improve fitness and well-being (4). While exercise therapy shows encouraging results for the treatment and prevention of adverse health outcomes in patients with chronic diseases, patients must adhere to the prescribed program in order to benefit from the exercise intervention (5). It is well-documented that long-term adherence to exercise therapy [i.e., adherence over a long period of time—lifelong—to control the disease (5)] is suboptimal in patients with chronic diseases and especially in home-based exercise therapy (2, 5). The WHO outlined that there are multiple factors underlying adherence, and that these factors can be classified in five dimensions affecting adherence in the general population; patient-related, social/economic, therapy-related, condition-related and health system factors (6). These five dimensions can provide an important framework (systems approach) for conceptualizing the issue of non-adherence with chronic diseases (7).

Non-adherence to exercise therapy, often exceeding 50% in patients with chronic diseases (5), is a problem which does not only effect the patient but also the health care system. Non-adherence entails high costs, both for patient and society, including avoidable morbidity, increased hospital admissions, and prolonged hospital stays (8). For example, a study in the Netherlands demonstrated that a 22% increase in adherence to exercise therapy as a first treatment strategy in Dutch patients with intermittent claudication (IC) resulted in an estimated 6% lower cost for IC treatment (9). To keep healthcare affordable and improving patient outcomes, focus on adherence is increasingly important (10).

To support successful implementation of home-based exercise programs for patients with chronic diseases, we must first identify what factors influence adherence to these programs. If these factors are known, this information can inform the design of future home-based exercise programs as well as the identification of individuals who may require extra support to benefit from prescribed exercise. Previous research identified a wide variety of prognostic factors potentially associated with adherence to physiotherapy (11, 12) and not to prescribed exercise therapy, or studied factors associated with adherence in older people, or studied a single aspect of exercise adherence (13). In addition, the results found are often inconsistent and sometimes contradicting, and quality of the studies is variable (14), leading to a poor understanding of the prognostic factors associated with the construct of adherence (15). To address this gap in the literature the aim of this systematic review was to identify and grade the quality of the evidence of variables associated with adherence to home-based exercise therapy in patients with chronic diseases.

## 2. Methods

### 2.1. Design

Methods comply with the Preferred Reporting Items for Systematic Reviews and Meta-Analyses (PRISMA) guidelines (16) (Supplementary Appendix S1) and with the registered protocol with the PROSPERO registration number CRD42021277003. All stages of the systematic review were conducted by two reviewers independently (ER, AD) using Covidence, a cloud-based systematic review platform. In case of disagreement a consensus meeting was scheduled. If discrepant judgements persisted, the judgement of a third author (EB) was decisive. Exceptions of this procedure are reported separately.

### 2.2. Search strategy, data sources and eligibility

The search strategy was developed in consultation with an information specialist (K.I. Sijtsma, Groningen University Medical Center). The following databases were searched from inception until August 1, 2022: PubMed, Embase, PsychINFO and CINAHL. The search strategy used for PubMed is outlined in Supplementary Appendix S2 and was translated to the remaining databases. No language restrictions were imposed. All reference lists of included studies were reviewed manually for eligible studies. In addition, grey literature sources (OpenGrey.eu, NARCIS.nl, DART-Europe.org, OATD.org) were searched using the term “exercise adherence”. We considered full text reports of cohort or cross-sectional studies and the experimental arm of randomized trials reporting prognostic factors associated with adherence to home-based exercise in patients (≥18 years) prescribed individual home-based exercise therapy for a chronic disease, i.e., conditions that last one year or more and require ongoing medical attention or limit activities of daily living or both (17). We considered all prognostic factors the authors associated with adherence. Adherence was defined as minutes of exercise completed or as number of sessions of exercise completed. For the outcome all validated and non-validated methods used for objectifying adherence were considered. If more than one publication was based on the same cohort or population reports were clustered (16).

### 2.3. Study selection

First we screened the title and abstract for potentially relevant studies, after Covidence automatically de-duplicated search results and facilitated further study selection (18). An exception on the two-reviewer process was protocoled if the search revealed more than 5,000 hits. In that case, the title/abstract round was assessed independently by ER and AD for the first 25% of the titles/abstracts. If there was less than 5% difference between the results (i.e., in at least 95% of cases, ER and AD came to the same conclusion regarding inclusion or exclusion), ER screened the remaining titles/abstracts (19). Otherwise, ER and AD both continued to perform the screening phase independently. Any study reviewed as “yes” or “unsure” was included to full-text review. When an abstract was identified but the full text was not available, we contacted the corresponding author *via* e-mail to obtain the full text. Next, we assessed the full text of all potentially relevant studies by applying the inclusion criteria.

### 2.4. Data extraction

For extracting data, we created a data extraction form based on the CHARMS checklist (20) in Covidence. Then, data was extracted by one reviewer (ER) and discussed with the other reviewer. Extracted data included information about the study: publication details (author, year, country), study design; the participants: sample size, gender and age, details of their chronic disease; information about the prognostic factors: studied variables associated with adherence with their effect estimates, and standard deviation or 95% confidence interval or *p*-values; and information about the outcome: used definition and measure of adherence. To structure the data, the identified prognostic factors were categorized into the five WHO-domains; (1) Patient-related, (2) Social/economic, (3) Therapy-related, (4) Condition-related, and (5) Health system factors (6) using the description of Sabatè (5). This categorization was chosen, because the WHO's systems model aims to analyze and provide explanations for non-adherence on a societal and health policy level in a broader sense. In this way, all identified prognostic factors could be placed in an appropriate domain (6).

In case relevant information was missing, we contacted authors *via* e-mail to obtain the missing information.

### 2.5. Risk of bias assessment

Risk of bias was assessed at the study level. Consistent with the Cochrane Collaboration’s recommendation, we used the Quality in Prognosis Studies (QUIPS) tool (21). The tool was used as described in the manual (21). Supplementary Appendix S3 provides an explanation on scoring of QUIPS.

### 2.6. Statistical analysis

#### 2.6.1. Data preparation

To enable pooling of all relevant studies, a single measure of effect size and an indication of the precision of the effect size were required. The most common effect size metric reported were odds ratios. Where another effect size was reported we converted this to the odds ratio metric (22).

Estimates were derived along with their 95% confidence intervals (CI) and *p*-values. In the absence of confidence intervals or standard errors, we calculated them using the formulas described by Altman and Bland (22). Where appropriate, direction of effect was converted for consistent reporting (i.e., showing associations between variables and non-adherence rather than adherence).

The primary analysis was structured in that the prognostic factors were already categorized into the five WHO-domains as described by Sabatè (5). A priori considerable clinical heterogeneity was expected in patients, specifically in type of chronic disease. Where relevant, this heterogeneity was addressed by making subgroups based on type of chronic disease (“cancer” vs. “other diseases”). Methodological heterogeneity was expected based on the variables associated with adherence assessed in the different included studies. After organizing the results, and pooling, we further explored possible sources of heterogeneity.

#### 2.6.2. Pooling methods

Where relevant, we protocoled a random-effects model to pool the overall effect of each prognostic factor found in one of the five domains. The pooling method used was the Inverse-variance, and the Paule-Mandel procedure to estimate the between-study heterogeneity (*τ*^2^) (22).

In case an original study did not provide adequate information to extract or calculate an effect size and relevant data could not be obtained from the authors, then this study could not be considered in the meta-analysis.

#### 2.6.3. Statistical heterogeneity

If pooling was possible, we assessed the outcome on statistical heterogeneity by eye-balling. Second, we calculated the Cochran Q as the weighted sum of squared differences between individual study outcomes and the pooled outcome across all studies. When *p* was significant (<0.05), statistical heterogeneity was considered to be present (23). Third, we used the *I*^2^ statistic to assess the variability between studies, the statistical heterogeneity. Low heterogeneity was considered with an *I*^2^ of less than 40%, moderate heterogeneity at 30%–60%, substantial heterogeneity at 50%–90% and considerable heterogeneity at 75%–100% (24). Because we considered a random-effects model effect sizes could show more variance than when drawn from a single homogeneous population. This between-study heterogeneity was quantified by using *τ*^2^.

#### 2.6.4. Subgroup- and sensitivity analysis

If statistical heterogeneity was considered to limit the interpretability of the pooled effect estimate, we explored the heterogeneity using subgroup analysis. If a study was identified as an outlier after eye-balling of the forest plots, we investigated that study further. Based on this, other subgroup analyses could be considered a posteriori to explain the observed heterogeneity.

Because we assumed a random-effects model within the subgroups, fixed-effects (plural) model (mixed-effects model) was considered. If the number of studies in the subgroups were small, we used a pooled version of *τ*^2^ across all subgroups. To determine whether a statistically significant subgroup difference was detected, we considered a *p*-value for this test of less than 0.1 to indicate a statistically significant subgroup effect. Furthermore, to interpret the subgroup analyses, we used the criteria of Richardson et al. (25).

We expected methodological heterogeneity in study design and methodological quality. Therefore, a sensitivity analysis was considered based on study design (“observational studies” vs. “other studies”) and/or risk of bias. We also considered a sensitivity analysis to examine what would happen if some aspect of the data or analysis were changed (23).

#### 2.6.5. Publication bias

We used funnel plots and Egger's test of small study effects (22) to assess the impact of possible publication bias when there were at least ten studies included in the meta-analysis. When there are fewer studies, the power of the test is considered too low to distinguish chance from real asymmetry. Funnel plots were assessed visually by eye-balling for asymmetry and statistically. A *p*-value <0.05 for the Egger's test indicated substantial asymmetry of the funnel plots, thereby implying possible publication bias (22).

### 2.7. GRADE

We used the GRADE prognostic factor framework to assign the strength and quality of evidence of association of prognostic factors with adherence. This process applies eight criteria that can upgrade or downgrade the quality of evidence supporting a prognostic factor and allows for evidence of a review of prognostic factors to be efficiently summarized for end-users (26). These criteria were applied as described by Huguet et al. (26). Prospective or retrospective cohort studies that test a fully developed hypothesis and conceptual framework without serious study limitations, and confirmatory studies without serious limitations constitute high-quality evidence on prognosis (26).

All statistical analyses were performed in R version 4.0.3. (27) using the {meta} package (28).

## 3. Results

### 3.1. Included studies

**Figure 1:**
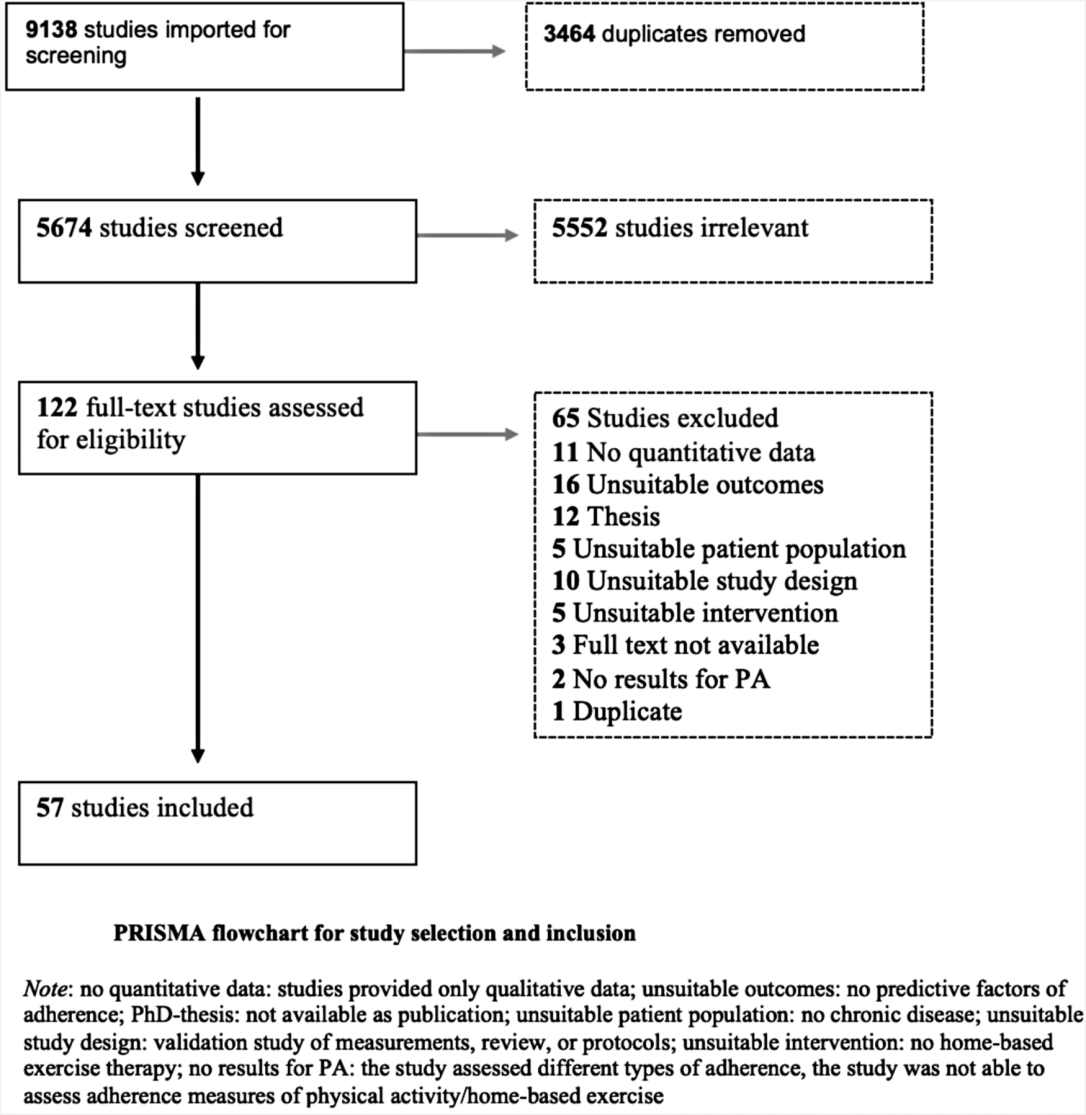
PRISMA flowchart for study selection and inclusion.

The PRISMA flowchart in Figure 1 shows the selection procedure. The search strategy initially identified 9,138 studies; 5,674 remained after duplicates were removed. Because there was less than 5% difference between the results of ER and AD, ER completed the title and abstract screening. Following title and abstract screening, 122 studies were assessed for full-text eligibility and 65 studies were excluded. The main reasons for exclusion were, no quantitative data, unsuitable study outcomes (no predictive factors of adherence), unsuitable study design (review or protocol) or it concerned a PhD-thesis of which no full text article was available or could not be obtained through the author (Figure 1 and Supplementary Appendix S4). We included the remaining 57 studies in this systematic review, of which two studies were translated from Korean into English (29, 30). No additional studies were identified through hand-searching or a search in the grey literature.

### 3.2. Study characteristics

Study characteristics are presented in Supplementary Appendix S5 and where applicable the set of covariates that was adjusted for are provided in Supplementary Appendix S6. Among the 57 included studies were 22 cross-sectional studies, 20 cohort studies, 10 randomized controlled trials, 4 secondary analysis and 1 database study. Countries of origin of studies included are Australia, Canada, USA, China, Turkey, Jordan, Poland, Sweden, Sudan, Hungry, Korea, Taiwan, Brazil, France, Germany, Pakistan and The Netherlands. A total of 29,541 individuals were prescribed home-based exercise across all studies (sample sizes ranged from 14 to 15,105 participants) and the average age ranged from 39 to 70 years. Chronic diseases included cardiovascular diseases (*n* = 24), cancer (*n* = 20), diabetes (*n* = 10), overweight/obesity (*n* = 1), chronic obstructive pulmonary disease (*n* = 1) and stroke (*n* = 1).

### 3.3. Adherence to prescribed exercise

In all studies, adherence was measured with self-reported questionnaires. Twenty-six studies used validated questionnaires, and the remaining studies used exercise logs, statements, or the simple question whether patients adhered to the program. The most commonly used questionnaire was the Godin Leisure Time Exercise Questionnaire (GLTEQ). A pedometer to objectify exercise was used by six studies (31–36).

### 3.4. Prognostic factors of exercise adherence

The starting point for the prognostic factors of exercise adherence were the five WHO domains. Patient-related factors were evaluated by 41 studies (29–32, 35, 37–72) (Table 1), social-economic factors by 29 studies (29–31, 33, 34, 43–45, 50, 51, 55, 57, 58, 61, 66, 69, 71–82), therapy-related factors by 5 studies (36, 45, 48, 72, 83), condition-related factors by 15 studies (34, 37, 42, 48, 51, 53, 61, 62, 71, 75, 78, 81–84) and health-system factors by 4 studies (48, 58, 61, 73).

**Table 1:** Summary of findings.

Outcomes	Effect measure (95% CI)	No. of participants (studies)	Quality of the evidence (GRADE)	Comments
Patient related prognostic factors of exercise adherence				
Self-efficacy	**OR 1.58** (1.27–1.97)	4,226 (18 studies)	⊗⊗⊗O Moderate^a^^,^^b^^,^^c^	More self-efficacy predicts better exercise adherence.
Exercise history	**OR 4.05** (1.10–14.90)	3,670 (10 studies)	⊗⊗⊗O Moderate^a^^,^^b^^,^^c^	Having a history of exercise predicts better exercise adherence.
Intention	**OR 1.47** (0.98–2.19)	864 (5 studies)	⊗⊗⊗O Moderate^a^^,^^b^^,^^c^	Intention is not predictive of exercise adherence.
Motivation	**OR 1.25** (1.12–1.39)	2,532 (5 studies)	⊗⊗⊗O Moderate^a^^,^^b^^,^^c^	Being more motivated predicts better exercise adherence.
Attitude	**OR 1.76** (0.69–4.48)	693 (2 studies)	⊗⊗⊗O Moderate^c^^,^^d^	Attitude is not predictive of exercise adherence.
PBC	**OR 1.21** (1.07–1.36)	215 (2 studies)	⊗⊗⊗⊗ High^c^^,^^e^	Having a higher PBC predicts better exercise adherence.
Perceived benefits	**OR 0.72** (0.13–4.05)	329 (2 studies)	⊗⊗⊗O Moderate^c^^,^^f^	Perceived benefits are not predictive of exercise adherence.
Social-economic factors of exercise adherence				
Education	**OR 2.07** (1.51–2.82)	19,705 (8 studies)	⊗⊗⊗O Moderate^a^^,^^c^	Higher education predicts better exercise adherence.
Social support	**OR 1.37** (0.92–2.05)	2,035 (8 studies)	⊗⊗OO Low^a^^,^^c^^,^^f^	Two studies reported more social support as a negative predictor of exercise adherence. The other studies showed more social support as a positive predictor.
Age	**OR 0.82** (0.57–1.20)	1,882 (5 studies)	⊗⊗OO Low^a^^,^^c^^,^^f^	Age is not predictive of exercise adherence.
Gender	**OR 3.17** (0.16–64.99)	1,506 (5 studies)	⊗⊗OO Low^a^^,^^c^^,^^f^	Gender is not predictive of exercise adherence.
Employment status	**OR 0.10** (0.00–7.69)	611 (4 studies)	⊗⊗OO Low^c^^,^^d^^,^^f^	Employment status is not predictive of exercise adherence.
Income	**OR 1.46** (0.96–2.21)	15,257 (2 studies)	⊗⊗OO Low^a^^,^^c^^,^^d^	Income is not predictive of exercise adherence.
Marital status	**OR 0.28** (0.01–8.74)	246 (2 studies)	⊗OOO Very Low^a^^,^^c^^,^^d^^,^^f^	Marital status is not predictive of exercise adherence.
Physical health	**OR 1.67** (1.20–2.31)	440 (2 studies)	⊗⊗OO Low^a^^,^^c^^,^^d^	Better physical health predicts better exercise adherence.
Therapy related prognostic factors of exercise adherence				
Duration of rehabilitation	**OR 1.06** (0.78–1.43)	109 (2 studies)	⊗⊗OO Low^a^^,^^c^^,^^f^	Duration of rehabilitation is not predictive of exercise adherence.
Condition related prognostic factors of exercise adherence				
BMI	**OR 0.54** (0.27–1.10)	733 (4 studies)	⊗OOO Very Low^a^^,^^c^^,^^d^^,^^f^	BMI is not predictive of exercise adherence.
Comorbidity	**OR 0.39** (0.21–0.72)	2,417 (2 studies)	⊗⊗⊗O Moderate^a^^,^^c^	Less comorbidities predicts better exercise adherence.
Depression	**OR 0.81** (0.72–0.91)	803 (3 studies)	⊗⊗OO Low^a^^,^^c^^,^^d^	Less depressive complaints predict better exercise adherence.
Fatigue	**OR 0.62** (0.41–0.94)	952 (4 studies)	⊗⊗OO Low^a^^,^^c^^,^^d^	Being less fatigued predict better exercise adherence.
Health-system related prognostic factors of exercise adherence				
No prognostic factors	–	–	–	–

CI, Confidence Interval; OR, odds ratio.

^a^ Phase 1 and 2 studies and hence downgraded by 1.

^b^ Significant heterogeneity among studies but all studies have same direction of effect and hence not downgraded.

^c^ Publication bias could not be assessed except for self-efficacy and exercise history, therefore not included the grading.

^d^ Serious limitations and hence downgraded by 1.

^e^ No limitations or inconsistency and hence not downgraded.

^f^ Inconsistency and hence downgraded by 1.

### 3.5 Quality assessment and GRADE recommendations

Risk of Bias per outcome/domain of the WHO is shown in traffic light plots in Supplementary Appendix S7. The overall Risk of Bias in patient-related factors was low RoB in 12 studies, moderate RoB in 5 studies and high RoB in 24 studies. In social-economic factors, 11 studies had low RoB, 3 studies had moderate RoB and 15 studies high RoB. In therapy-related factors, 3 studies had low RoB, 1 study had moderate RoB and 1 study had high RoB. In condition-related factors, 3 studies had low RoB, 2 studies had moderate RoB and 10 studies had high RoB. In health-system factors, 1 study had low RoB and 3 studies had high RoB. A concern regarding the quality of most included studies was the likelihood of selection bias and confounding.

Prognostic factors, categorized by the five domains, reported by at least two studies were assessed using the GRADE framework (Table 1). Publication bias could only be assessed for the prognostic factors self-efficacy and exercise history (more than ten studies); therefore, this was not included in the grading. Funnel plots for self-efficacy and exercise history are shown in Supplementary Appendix S8. The Egger's regression test showed statistically significant funnel plot asymmetry for the prognostic factor self-efficacy and exercise history (*p* < 0.0001). Also, visual inspection of the funnel plots by eye-balling suggested asymmetry and thus a potential publication bias.

Overall, higher exercise adherence was predicted by patient-related factors (more self-efficacy, exercise history, motivation and PBC), social-economic factors (better education and physical health) and condition-related factors (less comorbidities, depression and fatigue). The graphical presentations of the meta-analyses (forest plots) are presented in Supplementary Appendix S9.

### 3.6. Patient-related factors

**Figure 2:**
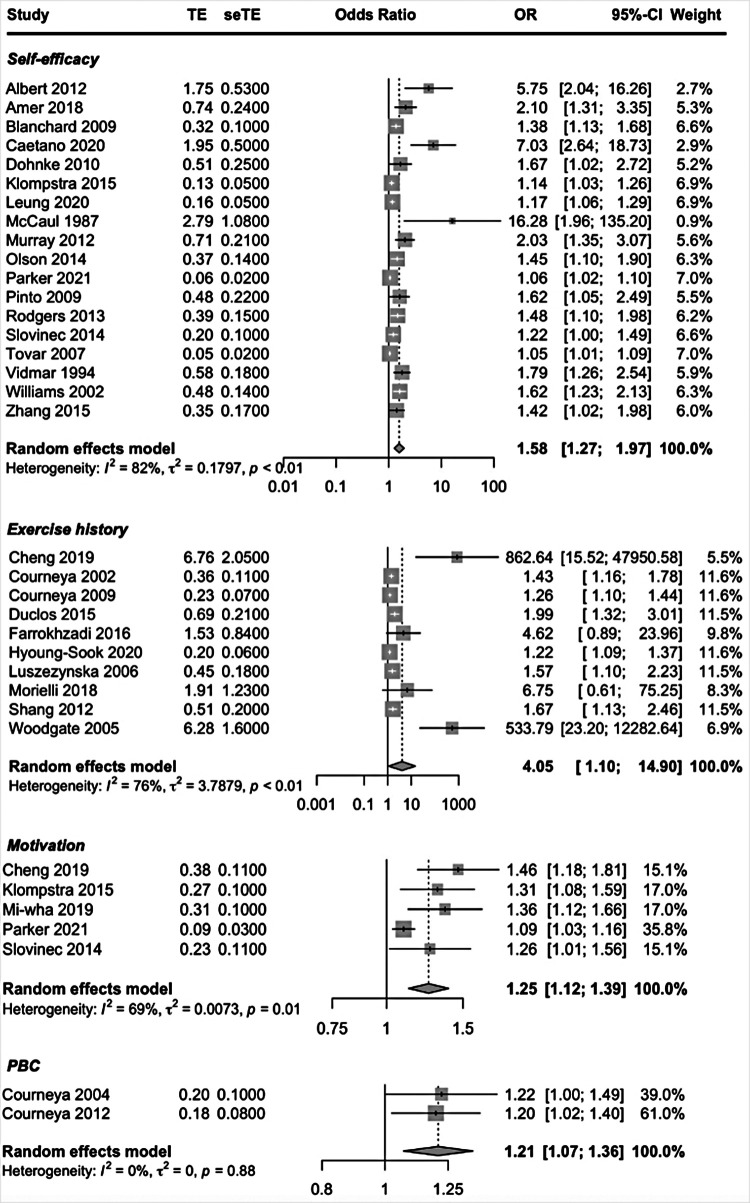
Forest plot patient-related factors.

Patient-related prognostic factors reported in more than one study included, self-efficacy, exercise history, intention, motivation, attitude, Perceived Behavioral Control (PBC) and perceived benefits. Higher self-efficacy, having an exercise history, motivation and PBC were the patient-related factors to be predictive of exercise adherence. High-quality evidence suggested that having higher PBC predicted higher exercise adherence. Moderate-quality evidence suggested that higher self-efficacy and having an exercise history predicted higher exercise adherence. The pooled ORs showed a significant better adherence rate; self-efficacy OR = 1.58 (95% CI, 1.27, 1.97; *I*^2^ = 82%), exercise history OR = 4.05 (95% CI, 1.10, 0; *I*^2^ = 76%), motivation OR = 1.25 (95% CI, 1.12, 1.39; *I*^2^ = 69% and PBC OR = 1.21 (95% CI, 1.07, 1.36; *I*^2^ = 0%) (Figure 2).

To address the heterogeneity, we performed subgroup analyses. When examining the covariates disease (cancer vs. other) and study design (cohort studies vs. other studies) for self-efficacy and exercise history a significant subgroup difference could not be found (self-efficacy *p* = 0.47 and *p* = 0.67; exercise history *p* = 0.15 and *p* = 0.51). Forest plots revealed three outliers in the prognostic factor self-efficacy; Albert et al. (37), McCaul et al. (35) and Caetano et al. (42), studies with, high, high and moderate risk of bias. In a sensitivity analysis these three studies were removed. By removing these three studies, subgroup differences remained not significant (*p* = 0.57 and *p* = 0.51). However, the *I*^2^ of the subgroup cohort studies went from 76% to 49%. The heterogeneity between studies is mainly due to the studies of Albert et al., McCaul et al. and Caetano et al.

The forest plot revealed two outliers in the prognostic factor exercise history: Cheng et al. (43) and Woodgate et al. (70), both studies with high RoB. By removing these studies heterogeneity dropped to *I*^2^ = 45% and subgroup differences remained not significant (*p* = 0.16 and *p* = 0.88), indicating that heterogeneity was mainly due to the studies of Cheng et al. and Woodgate et al.

When examining the covariate disease, a statistically significant subgroup difference (*p* < 0.01) was found for the prognostic factor motivation. However, the subgroup cancer included only one study, that of Parker et al. (63). The subgroup analysis by design gave no statistical difference between the two groups (*p* = 0.32), with the study of Parker et al. being the outlier (high RoB). Thus, heterogeneity could not be explained by subgroups, but by the study of Parker et al. The overall pooled effects seem robust enough to the influence of methodological and clinical heterogeneity in the studies and can be considered as study effects of this systematic review with some confidence. Forest plots of the subgroup- and sensitivity analyses are shown in Supplementary Appendix S10.

Moderate-quality evidence suggested that having the intention to exercise (OR = 1.47) and having a positive attitude (OR = 1.76) may be predictive of better exercise adherence, although not significant.

### 3.7. Social-economic factors

**Figure 3:**
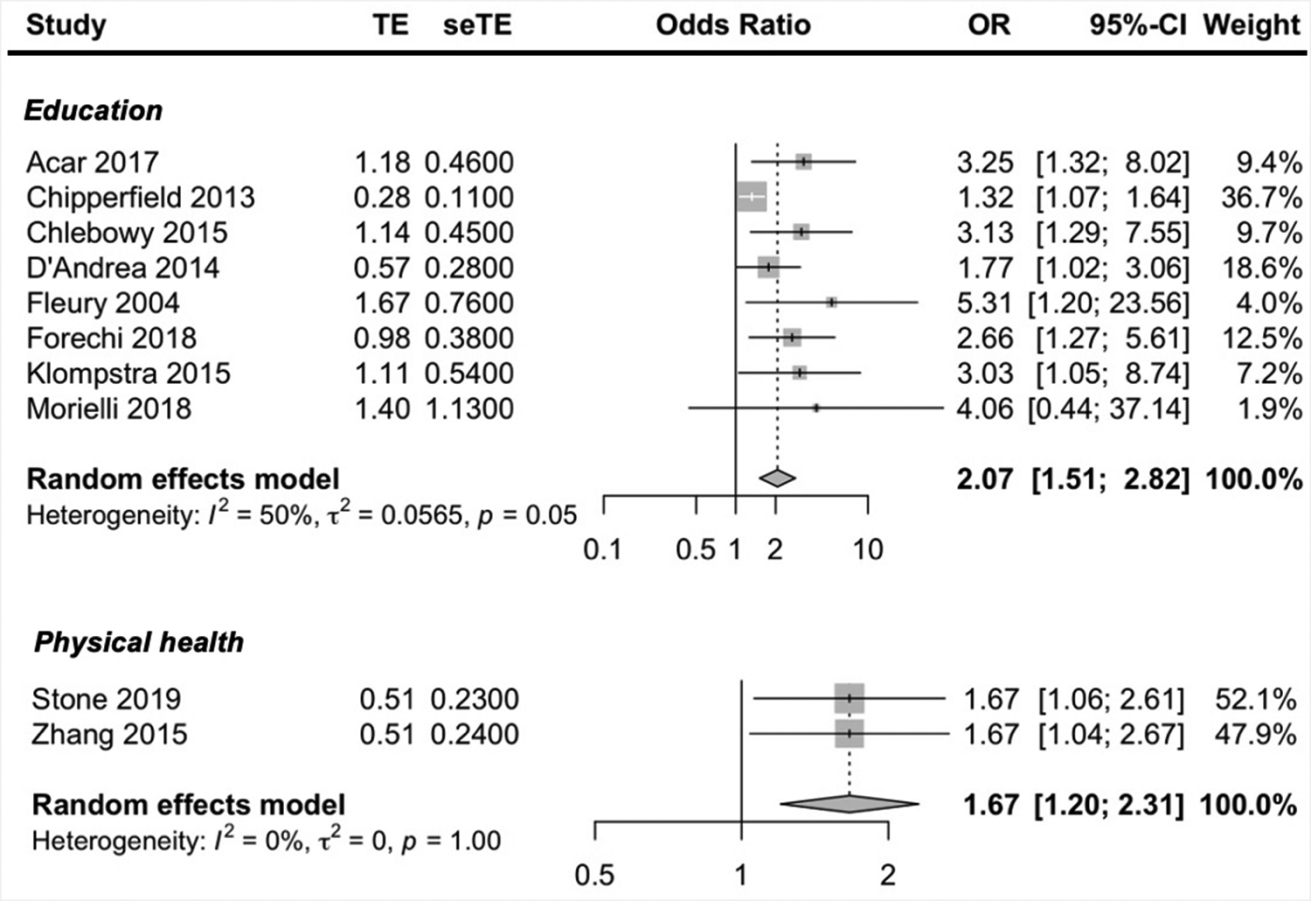
Forest plot social-economic factors.

Social-economic prognostic factors reported in more than one study included, education, social support, age, gender, employment status, income, marital status and physical health. Higher education and better physical health were the only social-economic factors to be predictive of exercise adherence. Moderate and low-quality evidence suggested that being higher educated and having a better physical health predicted higher exercise adherence. The pooled ORs showed a significant better adherence rate; education OR = 2.07 (95% CI, 1.51, 2.82; *I*^2^ = 50% and physical health OR = 1.67 (95% CI, 1.20, 2.31, *I*^2^ = 0%) (Figure 3).

To address heterogeneity in the prognostic factor education a subgroup analysis was performed on the covariate disease (Supplementary Appendix S10). A significant subgroup difference was found (*p* < 0.01), however the number of studies included in the analysis is small (three cancer and five other) but there was low unexplained heterogeneity (both cancer and other *I*^2^ = 0%), so there is some evidence to conclude that the type of chronic disease could explain the heterogeneity in the prognostic factor education; OR_cancer_ = 1.39 (95% CI, 1.14, 1.69; *I*^2^ = 0%) and OR_other_ = 3.11 (95% CI, 2.04, 4.74; *I*^2^ = 0%).

Low-quality evidence suggested that having more social support (OR = 1.37) may be predictive of better exercise adherence, although not significant and two studies (34, 58) reporting that more social support was predictive of lower exercise adherence.

### 3.8. Therapy-related factors

Therapy-related prognostic factors reported in more than one study included only duration of rehabilitation. However, this prognostic factor was not predictive of exercise adherence.

### 3.9. Condition-related factors

**Figure 4:**
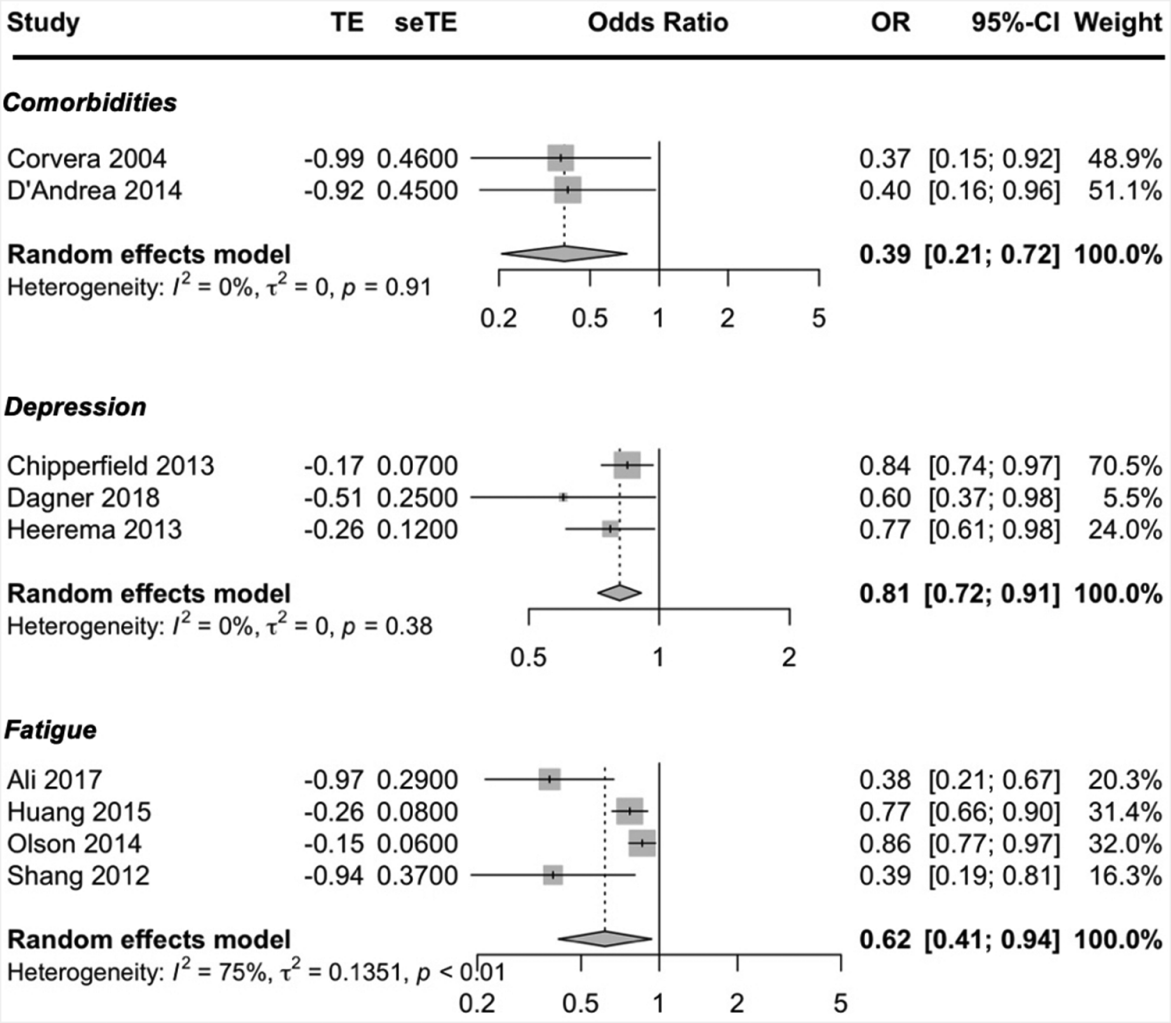
Forest plot condition-related factors.

Condition-related prognostic factors reported in more than one study included BMI, comorbidities, depression and fatigue. The condition-related factors to be predictive of exercise adherence were less comorbidities, less depressive symptoms and less fatigue. Moderate-quality evidence suggested that higher exercise adherence was predicted by having less comorbidities, less depressive symptoms and being less fatigued. The pooled ORs showed a significant better adherence rate; comorbidities OR = 0.39 (95% CI, 0.21, 0.72; *I*^2^ = 0%), depression OR = 0.81 (95% CI, 0.72, 0.91; *I*^2^ = 0%) and fatigue OR = 0.62 (95% CI, 0.41, 0.94; *I*^2^ = 75%) (Figure 4). Due to the small number of studies, a subgroup analysis could not be performed to address the heterogeneity in the prognostic factor fatigue (only one study was about cancer and only one study was a cohort study).

### 3.10. Health-system factors

In terms of health-system prognostic factors, no prognostic factors were reported by more than one study.

## 4. Discussion

### 4.1. Main findings

In this systematic review of prognostic factors of home-based exercise adherence in patients with chronic diseases, high-quality evidence supported that higher exercise adherence was predicted by the patient-related prognostic factor PBC. Moderate-quality evidence supported that higher exercise adherence was predicted by higher self-efficacy, having an exercise history and being motivated. Further, higher exercise adherence was predicted by the social-economic prognostic factors higher education (moderate-quality evidence) and better physical health (low-quality evidence). Also, higher exercise adherence was predicted by the condition-related prognostic factors, less comorbidities (moderate-quality evidence), less depressive symptoms (low-quality evidence) and being less fatigued (low-quality evidence).

### 4.2. Identified prognostic factors of adherence to home-based exercise

#### 4.2.1 Patient-related

Self-efficacy emerged from the review as a prognostic factor of adherence to home-based exercise. Self-efficacy has previously been reported as a prognostic factor of adherence in a systematic review of home-based physiotherapy (12) and is consistent with our findings. Also, the systematic review of Jack et al. (85) has reported that individuals with greater self-efficacy tended to be more adherent to outpatient physiotherapy. Greater self-efficacy, confidence in the ability to complete a given task, allows patients to overcome challenges with greater ease which seems especially important in home-based situations where there is no professional supervision (12).

Further, a history of exercise participation is a prognostic factor of home-based exercise adherence. This is in accordance with previous findings (12). If a patient has successfully completed similar behaviors before, this is likely to increase their perceptions of competence and therefore the likelihood of conducting the behavior again (86).

Also, higher exercise adherence was predicted by more motivation and more PBC, where PBC is seen as a similar construct as self-efficacy (87).

#### 4.2.2. Social-economic

Higher education also emerged as a prognostic factor of adherence to home-based exercise. This was supported with moderate-quality evidence based on phase one and two studies. Literature shows that higher rates of exercise and more frequent exercise participation have been correlated with increased education (88). Among adults over 65 years of age, education has not been found to be a significant prognostic factor of adherence in prospective exercise trials, yet larger longitudinal survey samples have indicated significant associations (88).

Social support was found as not predictive of home-based exercise adherence. This was a surprising finding because a common assumption is that patients who have more social support will be more adherent to exercise programs. Social support was reported as a strong prognostic factor of adherence to home-based physiotherapy (12). However, our finding was supported with low-quality evidence because of contradictory results and serious limitations. Therefore, this finding needs further investigation; who benefits from more social support where and when.

#### 4.2.3. Condition-related

The presence of less depressive symptoms predicted better adherence. This also has been found in previous research (12). Patients reporting feelings of depression were less likely to complete their exercises than those who did not have feelings of depression (89).

Two other condition-related prognostic factors of exercise adherence in this study were having less comorbidities and being less fatigued. Patients with less than two comorbidities and being less fatigued were more confident in the ability to complete their exercises.

When predicting exercise adherence, the most prognostic factors were found in the patient-related, social/economic and condition-related domains. Relatively little research has been conducted on the health-system factors and therapy-related factors of adherence. The common belief that patients are solely responsible for taking their treatment is misleading and most often reflects a misunderstanding of how other factors affect people's behavior and capacity to adhere to their treatment (5). Evidence available, might be biased by the traditional misconception that adherence is a patient-driven problem. This along with the fact that few predictors have been found, with low-quality evidence, suggests that follow-up research is needed to better understand and predict adherence in people with chronic diseases. This should also take into account the study design. Between-study heterogeneity is the rule rather than the exception in prognostic factor research (14). Longitudinal research designs are the only acceptable ones that provide prognostic evidence (26). Of the 57 studies included in this systematic review, 22 studies had a cross-sectional design. These designs can show correlations but are not the best design for examining predictive factors. Often a lot of factors are studied in relative small sample sizes, giving potentially high risk of bias (26).

### 4.3. Strengths and limitations

The study findings should be considered in the context of its strengths and limitations. The review was conducted predominantly according to best-practice methodologies, which included protocol pre-registration, development of the search strategy with help of an information specialist, review of multiple databases, a focus on adjusted estimates, no language restriction and contextualization of the findings within the GRADE strength of evidence framework, which benefits the generalizability of the findings of this systematic review.

The study has several limitations worth noting. First, despite the previously mentioned facts regarding study design for prognostic factor research, we chose to include both prospective cohort studies and cross-sectional studies in this systematic review. Our goal was not only to gather prognostic evidence, but also to gain insight into all possible variables that were studied. A risk is that the evidence may be downgraded by the cross-sectional studies, however, the subgroup analysis performed by design showed no subgroup differences between cohort studies and the other studies. Also, the level of evidence may have been downgraded because the risk of bias was assessed for all study designs using the QUIPS tool. For cross-sectional studies, the National Institutes of Health (NIH) Quality Assessment Tool for Observational Cohort and Cross- sectional studies is recommended (90). However, the use of two different tools may complicate the comparison of the risk of bias between studies. Since the signaling questions of both the QUIPS and the NIH have much overlap, we chose to use only the QUIPS tool. The risk of bias now compares well, only the QUIPS assesses cross-sectional studies more strictly than the NIH would. This may have slightly lowered the level of evidence as indicated by GRADE.

Second, only the lead researcher (ER) screened all the titles and abstracts, since there was less than 5% difference between the results in the first 25% of screening between ER and AD. This could have limited the validity of the screening process. Third, when undertaking a systematic review, there is always a risk of publication bias where negative studies of predictors not being associated with adherence might be less likely to get published. To reduce this problem a grey literature search of unpublished work was performed. None of the included studies were identified using this search strategy. Further, due to a power issue only of two prognostic factors a funnel plot could be created and the Egger's test for small study effects performed. So, publication bias was not included in the grading of the evidence. This may have influenced the quality of the evidence.

Finally, most of the studies did not use validated measures for assessing exercise adherence which limits the strength of their findings. This particular point is indicative of a wider issue around measuring exercise adherence.

### 4.4. Implications

The present study provides information on possible relevant prognostic factors of home-based exercise adherence in patients with chronic diseases and their respective effect sizes and can therefore be of help to develop better home-based exercise programs as well in the identification of individuals who may require extra support to benefit from prescribed home-based exercise therapy. Using the framework of WHO's five domains, it was confirmed that patients cannot be held solely responsible for their adherence. Factors external to the patient also play an important role in whether or not they are adherent to home-based exercise. Future programs and support should take this into account.

## 5. Conclusions

Design of prescribed home-based exercise programs for patients with chronic diseases requires an understanding of how characteristics of the patient and their environment impact exercise adherence. In this systematic review and meta-analyses, more precise risk estimates of known prognostic factors for home-based exercise adherence in patients with chronic diseases are provided. Based on the GRADE Framework for prognostic research, more PBC, higher self-efficacy, exercise history, higher education, better physical health, less comorbidities, less depressive symptoms, and less fatigue were the most important factors for predicting exercise adherence. These findings might aid in the development of future home-based exercise programs as well as in the identification of individuals who may require extra support to benefit from prescribed home-based exercise therapy.

## Data Availability

The original contributions presented in the study are included in the article/**Supplementary Material**, further inquiries can be directed to the corresponding author/s.
