# Supplementary material for: Prognostic factors of adherence to home-based exercise therapy in patients with chronic diseases: A systematic review and meta-analysis
Source: Front Sports Act Living. 2023 Mar 24;5:1035023. doi: 10.3389/fspor.2023.1035023 (PMC10080001; doi:10.3389/fspor.2023.1035023)
Supplement: Supplementary file 1 [file Datasheet1.docx]

**Addendum ‘Prognostic factors of adherence to home-based exercise therapy in patients with chronic diseases: a systematic review and meta-analysis’**

Contents

[Appendix 1: PRISMA Guidelines 2](#_Toc127525272)

[Appendix 2: Search strategy PubMed 6](#_Toc127525273)

[Appendix 3: Risk of bias explanation on scoring of QUIPS 7](#_Toc127525274)

[Appendix 4: Full text review reasons for exclusion 9](#_Toc127525275)

[Appendix 5: Study characteristics 13](#_Toc127525276)

[Appendix 6: Covariates within multivariable and adjusted models 32](#_Toc127525277)

[Appendix 7: Risks of bias traffic light plots per domain 35](#_Toc127525278)

[Appendix 8: Publication bias funnel plots 38](#_Toc127525279)

[Appendix 9: Forest plots per domain 39](#_Toc127525280)

[Appendix 10: Subgroup- and sensitivity analysis 44](#_Toc127525281)

# **Appendix 1: PRISMA Guidelines**

| **Section and Topic** | **Item #** | **Checklist item** | **Reported on page #** |
| --- | --- | --- | --- |
| **TITLE** | | |  |
| Title | 1 | Identify the report as a systematic review. | 1 |
| **ABSTRACT** | | |  |
| Abstract | 2 | See the PRISMA 2020 for Abstracts checklist. | 1 |
| **INTRODUCTION** | | |  |
| Rationale | 3 | Describe the rationale for the review in the context of existing knowledge. | 2 |
| Objectives | 4 | Provide an explicit statement of the objective(s) or question(s) the review addresses. |  |
| **METHODS** | | |  |
| Eligibility criteria | 5 | Specify the inclusion and exclusion criteria for the review and how studies were grouped for the syntheses. | 3/Appendix 3 |
| Information sources | 6 | Specify all databases, registers, websites, organisations, reference lists and other sources searched or consulted to identify studies. Specify the date when each source was last searched or consulted. | 3 |
| Search strategy | 7 | Present the full search strategies for all databases, registers and websites, including any filters and limits used. | 3/Appendix 2 |
| Selection process | 8 | Specify the methods used to decide whether a study met the inclusion criteria of the review, including how many reviewers screened each record and each report retrieved, whether they worked independently, and if applicable, details of automation tools used in the process. | 3 |
| Data collection process | 9 | Specify the methods used to collect data from reports, including how many reviewers collected data from each report, whether they worked independently, any processes for obtaining or confirming data from study investigators, and if applicable, details of automation tools used in the process. | 3, 4 |
| Data items | 10a | List and define all outcomes for which data were sought. Specify whether all results that were compatible with each outcome domain in each study were sought (e.g. for all measures, time points, analyses), and if not, the methods used to decide which results to collect. | 3/Appendix 4, 5 |
|  | 10b | List and define all other variables for which data were sought (e.g. participant and intervention characteristics, funding sources). Describe any assumptions made about any missing or unclear information. | 3 |
| Study risk of bias assessment | 11 | Specify the methods used to assess risk of bias in the included studies, including details of the tool(s) used, how many reviewers assessed each study and whether they worked independently, and if applicable, details of automation tools used in the process. | 4/Appendix 6 |
| Effect measures | 12 | Specify for each outcome the effect measure(s) (e.g. risk ratio, mean difference) used in the synthesis or presentation of results. | 4 |
| Synthesis methods | 13a | Describe the processes used to decide which studies were eligible for each synthesis (e.g. tabulating the study intervention characteristics and comparing against the planned groups for each synthesis (item #5)). | 4 |
|  | 13b | Describe any methods required to prepare the data for presentation or synthesis, such as handling of missing summary statistics, or data conversions. | 4, 5 |
|  | 13c | Describe any methods used to tabulate or visually display results of individual studies and syntheses. | 5 |
|  | 13d | Describe any methods used to synthesize results and provide a rationale for the choice(s). If meta-analysis was performed, describe the model(s), method(s) to identify the presence and extent of statistical heterogeneity, and software package(s) used. | 5 |
|  | 13e | Describe any methods used to explore possible causes of heterogeneity among study results (e.g. subgroup analysis, meta-regression). | 5 |
|  | 13f | Describe any sensitivity analyses conducted to assess robustness of the synthesized results. | 5 |
| Reporting bias assessment | 14 | Describe any methods used to assess risk of bias due to missing results in a synthesis (arising from reporting biases). | 5 |
| Certainty assessment | 15 | Describe any methods used to assess certainty (or confidence) in the body of evidence for an outcome. | 6 |
| **RESULTS** | | |  |
| Study selection | 16a | Describe the results of the search and selection process, from the number of records identified in the search to the number of studies included in the review, ideally using a flow diagram. | 6 |
|  | 16b | Cite studies that might appear to meet the inclusion criteria, but which were excluded, and explain why they were excluded. | 6 |
| Study characteristics | 17 | Cite each included study and present its characteristics. | 6/Appendix 3 |
| Risk of bias in studies | 18 | Present assessments of risk of bias for each included study. | 7/Appendix 8 |
| Results of individual studies | 19 | For all outcomes, present, for each study: (a) summary statistics for each group (where appropriate) and (b) an effect estimate and its precision (e.g. confidence/credible interval), ideally using structured tables or plots. | 7, 8, 9/Appendix 7 |
| Results of syntheses | 20a | For each synthesis, briefly summarise the characteristics and risk of bias among contributing studies. | 7, 8, 9 |
|  | 20b | Present results of all statistical syntheses conducted. If meta-analysis was done, present for each the summary estimate and its precision (e.g. confidence/credible interval) and measures of statistical heterogeneity. If comparing groups, describe the direction of the effect. | 7, 8, 9 |
|  | 20c | Present results of all investigations of possible causes of heterogeneity among study results. | 7, 8, 9 |
|  | 20d | Present results of all sensitivity analyses conducted to assess the robustness of the synthesized results. | 7, 8, 9 |
| Reporting biases | 21 | Present assessments of risk of bias due to missing results (arising from reporting biases) for each synthesis assessed. | 7/Appendix 9 |
| Certainty of evidence | 22 | Present assessments of certainty (or confidence) in the body of evidence for each outcome assessed. | 7, 8, 9 |
| **DISCUSSION** | | |  |
| Discussion | 23a | Provide a general interpretation of the results in the context of other evidence. | 10, 11 |
|  | 23b | Discuss any limitations of the evidence included in the review. | 11, 12 |
|  | 23c | Discuss any limitations of the review processes used. | 11, 12 |
|  | 23d | Discuss implications of the results for practice, policy, and future research. | 12 |
| **OTHER INFORMATION** | | |  |
| Registration and protocol | 24a | Provide registration information for the review, including register name and registration number, or state that the review was not registered. | 1 |
|  | 24b | Indicate where the review protocol can be accessed, or state that a protocol was not prepared. | 1 |
|  | 24c | Describe and explain any amendments to information provided at registration or in the protocol. | Not applicable |
| Support | 25 | Describe sources of financial or non-financial support for the review, and the role of the funders or sponsors in the review. | 15 |
| Competing interests | 26 | Declare any competing interests of review authors. | 15 |
| Availability of data, code and other materials | 27 | Report which of the following are publicly available and where they can be found: template data collection forms; data extracted from included studies; data used for all analyses; analytic code; any other materials used in the review. | 15 |

*From:*  Page MJ, McKenzie JE, Bossuyt PM, Boutron I, Hoffmann TC, Mulrow CD, et al. The PRISMA 2020 statement: an updated guideline for reporting systematic reviews. BMJ 2021;372:n71. doi: 10.1136/bmj.n71

For more information, visit: <http://www.prisma-statement.org/>

# **Appendix 2: Search strategy PubMed**

("Chronic Disease"[Mesh] OR chronic diseas*[tiab] OR "Noncommunicable Diseases"[Mesh] OR Noncommunicable Diseas*[tiab] OR non-communicable diseas*[tiab] OR chronic ill*[tiab] OR chronically ill*[tiab] OR Chronic health condition*[tiab] OR Chronic health issue*[tiab] OR "Pulmonary Disease, Chronic Obstructive"[Mesh] OR Chronic obstructive pulmonary diseas*[tiab] OR COPD[tiab] OR "Diabetes Mellitus"[Mesh] OR Diabetes[tiab] OR "Obesity"[Mesh] OR Obes*[tiab] OR "Depression"[Mesh] OR Depress*[tiab] OR "Cardiovascular Diseases"[Mesh] OR Cardiovascular diseas*[tiab] OR "Neoplasms"[Mesh] OR Cancer*[tiab] OR neoplasm*[tiab])

AND

("Exercise Therapy"[Mesh] OR "Physical Therapy Modalities"[Mesh] OR physical activit*[tiab] OR exercis*[tiab] OR physical therap*[tiab])

AND

("Treatment Adherence and Compliance"[Mesh] OR "Patient Compliance"[Mesh] OR Adheren*[tiab] OR Non-adheren*[tiab] OR Complian*[tiab] OR Non-complian*[tiab] OR Self-manag*[tiab])

AND

(Barrier*[tiab] OR Facilita*[tiab] OR Enabler*[tiab] OR predict*[tiab]) NOT (("Child"[Mesh] OR "Adolescent"[Mesh] OR "Infant"[Mesh]) NOT "Adult"[Mesh])

# **Appendix 3: Risk of bias explanation on scoring of QUIPS**

**Q1: Study participation**

Low risk: Population of interest, description of inclusion and exclusion criteria, description of time and place of recruitment and adequate description of baseline study sample.

Moderate risk: adequate description of population of interest, some missing data about inclusion and exclusion criteria.

High risk: extreme selection or population of interest is not described (no inclusion or exclusion criteria, no description of time and place of recruitment).

**Q2: Study Attrition**

Low risk: Documentation of follow-up and actions are undertaken to retrieve information

from these cases.

Moderate risk: Intermediate.

High risk: No information or mentioning on loss-to follow-up.

**Q3: Prognostic factor (PF) Measurement**

Low risk: Definition of PF is provided and reproducible. The risk of misclassification is

extremely low.

Moderate risk: Definition is given, but not specific.

High risk: No clear definition given for all factors.

**Q4: Outcome Measurement**

Low risk: Definition of outcome is provided and reproducible. The risk of misclassification is

extremely low.

Moderate risk: Definition is given, but reproducibility is questionable.

High risk: No clear definition of outcome is given or process of collection information on

occurrence of outcomes is not described.

**Q5: Study Confounding (Covariates)**

Low risk: Most important covariates were evaluated and the result was either adjusted for

these variables or reported in a multivariable model.

Moderate risk: Some important covariates were unadjusted for or missing in the cohort.

High risk: Covariates were not measured

**Q6: Statistical analysis and Reporting**

Low risk: A fitting analysis was undertaken (e.g. conditional logistic regression in the setting

of a case-control design); steps in model building were undertaken well and adequate

reporting (univariable as well as multivariable).

Moderate: Only univariable data are provided in a study in which a multivariable model could

have been performed.

High risk: OR could not be calculated because there was insufficient data (e.g. only a p value).

# **Appendix 4: Full text review reasons for exclusion**

| **Title** | **Author (year)** | **Reason for exclusion** |
| --- | --- | --- |
| Relationships among health beliefs, self-efficacy, and exercise adherence in patients with coronary artery disease | Robertson (1992) | Thesis |
| Exercise maintenance following pulmonary rehabilitation: effect of distractive stimuli | Bauldoff (2002) | Wrong outcomes |
| The predictors of non-adherence of regular physical activity after coronary revascularization | Ipek 2014 | Duplicate |
| The effects of compliance with exercise training on pulmonary rehabilitation | Sassi-Dambron (1994) | Wrong outcomes |
| [Factors associated with adherence to physical activity in patients with chronic non-communicable diseases] | Quiroz-Mora (2018) | Full text not available |
| Locus of control, self-efficacy and adherence to medical regime in diabetic patients | Kadambari (2018) | Full text not available |
| Self-management strategies in overweight and obese Canadians with arthritis | Bernatsky (2012) | Wrong outcomes |
| Maintenance of exercise behavior for individuals at risk for cardiovascular disease | Sorensen (1997) | Wrong patient population |
| Predictors of physical activity at 12month follow-up after a supervised exercise intervention in postmenopausal women | Aparicio-Ting (2015) | Wrong patient population |
| Self reported adherence to a home-based exercise programme among people with Parkinson's disease | Pickering (2013) | Wrong patient population |
| Role of psychosocial factors in long-term adherence to secondary prevention measures after myocardial infarction: a longitudinal analysis | Nachshol (2020) | Wrong study design |
| Support from next of kin and nurses are significant predictors of long-term adherence to treatment in post-PCI patients | Kähkönen (2020) | Wrong outcomes |
| Barriers to cardiac rehabilitation: DOES AGE MAKE A DIFFERENCE? | Grace (2009) | Wrong outcomes |
| Participation in and adherence to physical exercise after completion of primary cancer treatment | Kampshoff (2016) | Wrong intervention |
| Physical activity preferences among patients with lung cancer in Taiwan | Lin (2013) | Wrong outcomes |
| Development of an exercise adherence program for breast cancer survivors with cancer-related fatigue-an intervention mapping approach | Kim (2019) | Wrong outcomes |
| Predictors of cancer survivors' response to a community-based exercise program | Brunet (2020) | Wrong intervention |
| Is Physical Activity a Risk or Protective Factor for Subsequent Dietary Lapses Among Behavioral Weight Loss Participants? | Crochiere (2020) | Wrong study design |
| Physical activity in women receiving chemotherapy for breast cancer: patterns and adherence to an intervention protocol | Swenson (2006) | Thesis |
| Factors influencing adherence to home-based exercises among community-dwelling stroke survivors in India: a qualitative study | Mahmood (2021) | Wrong outcomes |
| Long-term exercise adherence after public health training in at-risk adults | Saida (2017) | Wrong patient population |
| A beginning look at the effect of age on dyspnea, physical functioning and self-efficacy for home walking and managing shortness of breath in adults with chronic obstructive pulmonary disease (COPD) | Mackin (2007) | Thesis |
| Predictors of compliance with diet and exercise six months after heart transplantation | Grady (1993) | Thesis |
| A practical use of theory to study adherence | Granger (2007) | Wrong study design |
| Self-Monitoring Using Continuous Glucose Monitors with Real-Time Feedback Improves Exercise Adherence in Individuals with Impaired Blood Glucose: A Pilot Study | Bailey (2016) | Wrong study design |
| Predictors of Adherence to Self-Care in Rural Patients With Heart Failure | Biddle (2020) | No results for PA |
| Exercise after Stroke: Patient Adherence and Beliefs after Discharge from Rehabilitation | Miller (2017) | Full text not available |
| Characteristics Predictive of Lifestyle Change Among Older Adults with Hypertension | Douglas (2012) | Thesis |
| Understanding the barriers to physical activity for cancer patients: review and recommendations | Brawley (2002) | Wrong study design |
| The effects of telephone contact on the exercise adherence of cardiac patients | Hopper (1995) | Thesis |
| Exercise adherence and contamination in a randomized control trial of a home-based walking program among patients receiving active cancer treatment | Shang (2009) | Thesis |
| Who listens to our advice? Predictors of adherence to self-care recommendations in rural patients with heart failure | Biddle (2013) | No results for PA |
| Research for practice. Women's inhibitors and facilitators associated with making behavioral changes after myocardial infarction | McSweeney (2004) | Wrong intervention |
| A mobile phone-based care model for outpatient cardiac rehabilitation: the care assessment platform (CAP) | Walters (2010) | Wrong study design |
| Barriers and enablers of physical activity engagement for patients with COPD in primary care | Kosteli (2017) | Wrong intervention |
| Determinants of adherence in women managing chronic health conditions | Nichols (2004) | Thesis |
| Why Don't Our Patients with Chronic Obstructive Pulmonary Disease Listen to Us? The Enigma of Nonadherence | Blackstock (2016) | Wrong study design |
| Personal factors, perceptions, influences and their relationship with adherence behaviors in patients with diabetes | Hagerstrom (2010) | Thesis |
| Prediction of the Adherence to a Home-Based Cardiac Rehabilitation Program | Claes (2019) | Wrong outcomes |
| The relationship between self-efficacy, internal health locus-of-control and adherence with a therapeutic diabetes regimen | Prendergast (1993) | Thesis |
| Enhancing Exercise Adherence for Breast Cancer Survivors | Pekmezi (2012) | Wrong study design |
| Adherence to Self-Care Practices and Associated Factors Among Outpatient Adult Heart Failure Patients Attending a Cardiac Center in Addis Ababa, Ethiopia in 2020 | Tegegn (2021) | Wrong outcomes |
| Differences in physical activity domains, guideline adherence, and weight history between metabolically healthy and metabolically abnormal obese adults: a cross-sectional study | Kanagasabai (2015) | Wrong intervention |
| The exercise protocol adherence of cancer patients in a randomized controlled trial | Chen (2019) | Wrong outcomes |
| Exercise as an intervention for cancer-related fatigue: The subject's view | Coon (2003) | Wrong study design |
| Comparison between hybrid and standard centre-based cardiac rehabilitation in female patients after myocardial infarction: a pilot study | Korzeniowska-Kubacka (2014) | Wrong outcomes |
| Facilitators and barriers to heart failure self-care | Riegel (2002) | Wrong outcomes |
| Adherence to exercise in patients with chronic obstructive pulmonary disease | Cuenco (2003) | Thesis |
| Predictors of exercise relapse in individuals with cardiovascular disease | Wilkinson (2003) | Wrong outcomes/thesis |
| Predictors of short- and long-term adherence to a daily walking program in persons With Alzheimer's disease | McCurry (2010) | Wrong patient population |
| Influence of Social Support and Negative Emotional Status on Self-care Adherence in Symptomatic Patients with Heart Failure | 양인 (2016) | Wrong outcomes |
| Stories of exercise noncompliance among patients with chronic obstructive pulmonary disease after completion of pulmonary rehabilitation | Rabinowitz (1999) | Thesis |
| The effectiveness of health education using the teach-back method on adherence and self-management in chronic disease: a systematic review protocol | ThiThuyHa (2013) | Wrong study design |

# **Appendix 5: Study characteristics**

**Table 1 Study characteristics**

A. Quantitative studies

| **Author** | **Study design** | **N** | **% Male** | **Age (SD)** | **Disease** | **Adherence** | **Effect size univariable** | **Effect size multivariable** | **Domain** |
| --- | --- | --- | --- | --- | --- | --- | --- | --- | --- |
| Vidmar 1994 (US) | Cross sectional | 138 | 85.5 | 45-64 (range) | Cardiovascular | Exercise Compliance Questionnaire | Self-efficacy B=0.581, p=0.001 | - | 1 |
| Parker 2021 (US) | Cross sectional | 262 | 55.3 | 68 (range 30-93) | Pancreatic cancer | Modified GLTEQ | - | Motivation OR=1.09, 95%CI=1.03-1.16, p=0.004;  Self-efficacy OR=1.06, 95%CI=1.02-1.10, p=0.001 | 1  1 |
| Leung 2020 (China) | Mixed-method (cohort) | 140 | 17.1 | 39.2 ± 10.5 | Overweight and obesity | Program PA score and IPAQ score | - | Self-efficacy B=0.16, 95%CI=0.07-0.24, p<0.001;  Stage of change B=0.83, 95%CI=0.07-1.58, p=0.031 | 1  1 |
| Cheng 2019 (China) | Cohort | 1033 | 73.3 | 55.96 ± 10.50 | Coronary heart disease | HEAQ questionnaire | - | Social support B=0.487, 95%CI=0.4111-0.563, p<0.001;  Age B=0.337, 95%CI=0.182-0.491, p<0.001;  Gender B=8.318, 95%CI=4.620-12.017, p<0.001;  Exercise history B=6.756, 95%CI=3.471-10.041, p<0.001  Motivation B=0.376, 95%CI=0.264-0.488, p<0.001 | 2  2  2  1  1 |
| Albert 2012 (US) | Prospective, correlational (cohort) | 492 | 64.8 | 63 ± 13.6 | Chronic heart failure | Moderate-vigorous exercise | - | Perception of good health OR=8.57, 95%CI=4.-0-19.18  Self-confidence for exercise OR=5.74, 95%CI=3.8-8.95;  Barriers OR=3.41, 95%CI=2.36-5.06;  Attitude OR=3.01, 95%CI=2.2-4.2; Perceived value OR=2.58, 95%CI=1.83-3.71;  Benefits/motivators OR=2.25, 95%CI=1.64-3.15;  Knowledge OR=1.26, 95%CI=1.01-1.57 | 4  1  1  1  1  1  1 |
| Acar 2015 (Turkey) | Cohort | 202 | 73% | 61.3 ± 11.2 | Coronary revascularization | PA status (regular exercise) as described by the intervention **(non-adherence)** | - | Education OR=3.26, 95%CI=1.31-8.11, p=0.01  Regular follow-up OR=2.95, 95%CI=1.01-8.61, p=0.04 | 2  5 |
| Woodgate 2005 (Canada) | Cohort | 64 | 92.2% | 65 ± 10.14 | Myocardial infarction | Exercise log and BORG scale | - | Past exercise R^2^=0.75, p<0.0001 | 1 |
| Williams 2002 (Australia) | Cross sectional | 94 | 43.6% | 62.2 ± 12 | Diabetes | SDSCA and TAM (the exercise questions) | - | Self-efficacy B=0.48, p<0.001 | 1 |
| Tovar 2007 (US) | Cross sectional | 212 | 33% | 58 (range 25-85) | Diabetes | TDAQ | - | Self-efficacy B=0.053, p=0.034 | 1 |
| Tooth 1993 (US) | Cohort | 29 | 100% | 66.3 ± 7.69 | Myocardial infarction | Exercise logbook | - | Occupation p<0.05; Expectations p<0.05 | 2  1 |
| Wilson 1986 (US) | Cross sectional | 184 | 33.2% | 57.9 ± 10.2 | Diabetes | Diabetes-Specific Assessment Battery | - | Health beliefs r=0.40, p<0.001; Social support r=0.24, p<0.01 | 1  2 |
| Mosleh 2015 (Jordan) | Cross sectional | 254 | 56.9% | 52 ± 15.6 | Coronary heart disease | GLTEQ | - | Age B=-0.45, 95%CI=-0.71:-0.19, p<0.001;  BMI B=-1.41, 95%CI=-2.2:-0.64, p<0.001 | 2  4 |
| Miller 1990 (US) | RCT | 51 | 78% | 55 | Myocardial infarction | HBS | - | Perceived beliefs of others B=0.441, p<0.002 | 2 |
| McGuire 2011 (US) | Secondary analysis of exercise group RCT | 120 | 0% (all women) | 58.7 | Breast cancer (survivors) | Exercise log | - | Previous adherence B=0.31, p<0.001;  Marital status B=0.10, p=0.030; Providing feedback B=0.40, p<0.001;  Providing support B=-0.27, p<0.001  Promoting knowledge and skills B=-0.136, p=0.032 | 1  2  5  5  5 |
| Luszczynska 2006 (Poland) | Cohort | 114 | 64% | 54.3 ± 6.90 | Myocardial infarction | Asking about performance of the recommended exercises | - | PA before MI OR=1.57, 95%CI=1.12-2.21, p<0.01;  Intention OR=3.46, 95%CI=1.13-10.61, p<0.05; Perceived support OR=10.78, 95%CI=2.00-58.23, p<0.01 | 1  1  2 |
| Dagner 2019 (Sweden) | Cross sectional | 368 | unknown | 63 ± 8.15 | Myocardial infarction | Amount of PA per week (**non-adherence**) | Mobility problems OR=2.4, 95%CI=1.0-5.4; Pain/discomfort OR=2, 95%CI=1.2-3.6; Anxiety/depression OR=0.6, 95%CI=0.3-1.0; Age OR=0.9, 95%CI=0.9-1.0 | - | 4  4  4  2 |
| Amer 2018 (Sudan) | Cross sectional | 392 | 54.1% | <50 years (36.2%) | Diabetes | Exercise log | Self-efficacy OR=2.1, 95%CI=1.3-5.0, p=0.002 | - | 1 |
| Zhang 2015 (Canada) | Cross sectional | 40 | 77.0% | 66.2 ± 10.0 | Heart failure | SCHFI V6.2 | BMI B=-0.77, p=0.01; Employment status B=-6.65, p=0.02;  Physical health B=0.51, p=0.03 | - | 4  2  2 |
| SlovinecD’angelo 2014 (Canada) | Cohort | 801 | 75.4% | 61.4 ± 10.0 | Coronary heart disease | GLTEQ | Autonomous motivation X^2^=3.93, p<0.05; Self-efficacy X^2^=2.83, p<0.05 | - | 1  1 |
| Shang 2012 (US) | Secondary analysis of RCT | 126 | 61.1% | 60.2 ± 10.5 | Cancer | Exercise log and pedometer | Baseline physical fitness B=0.51, p<0.01; Pretreatment fatigue level B=-0.93, p<0.01; Mood-disturbance B=-0.27, p<0.01;  Marital status B=-3.56, p<0.05 | - | 1  4  4  2 |
| Rodgers 2013 (Canada) | Cohort | 114 | 78.1% | 57.7 ± 9.8 | Cardiovascular disease | Modified version of GLTEQ | Self-efficacy B=0.39, p<0.01 | - | 1 |
| Pinto 2009 (US) | RCT | 43 | 0% (all women) | 53.42 ± 9.08 | Breast cancer | 7-day PAR, exercise logs and pedometers | - | Self-efficacy OR=1.61, p=0.03 | 1 |
| Olson 2014 (US) | Cross sectional | 483 | 0% (all women) | 63 ± 12 | Breast cancer | GLTEQ | - | Fatigue B=-0.15, p<0.01;  Self-efficacy B=0.37, p<0.001; Perceived barriers B=-0.18, p<0.05 | 4  1  1 |
| Ng 2021 (US) | Cross sectional | 200 | 45% | unknown | Cancer | Stanford Patient Education Research Center Exercise Behaviors Survey | - | Enjoyment OR=7.064, p=0.0029; Symptom burden OR=0.7311, p=0.006; Functional score OR=1.051, p=0.016;  Support from physician OR=4.702, P=0.0082;  Gender OR=0.135, p=0.002 | 1  4  4  5  2 |
| Murray 2012 (Canada) | Cohort | 129 | 85.3% | 63.45 ± 9.78 | Coronary vascular disease | Modified version GLTEQ | - | Self-efficacy B=0.71, p=0.00 | 1 |
| Morielli 2018 (Canada) | Cohort | 17 | 70.6% | <60 years (41.2%) | Rectal cancer | GLTEQ | Mental health r=-0.56, p-0.046; Gender d=0.54, p=0.38;  Education d=0.77, p=0.22;  Exercise at baseline d=1.05, p=0.12 | - | 4  2  2  1 |
| Moore 2003 (US) | Cohort | 60 | 0% (all women) | 64.6 ± 10.3 | Myocardial infarction | Portable wristwatch heart rate monitors | - | Social support B=0.30, p=0.02 | 2 |
| McNeil 2021 (Canada) | RCT | 30 | 0% (all women) | 57.7 ± 9.6 | Breast cancer | Polar A360 activity tracker | - | Social support OR=0.83, 95%CI=0.72-0.97; Baseline VO_2max_ OR=0.98, 95%CI=0.95-0.99 | 2  4 |
| McCaul 1987 (US) | Cohort | 107 | 46.7% | 31.3 (range 12-65) | Insulin-dependent diabetes | Self-monitored PA and activity monitor | Self-efficacy correlation=0.61, p<0.01 | - | 1 |
| Klompstra 2015 (Sweden) | Cross sectional | 154 | 68,2% | 70 ± 10 | Heart failure | s-IPAQ | Education p=0.04; Self-efficacy mean 2 ± 1, p<0.01; Motivation mean 4 ± 2, p<0.01 |  | 2  1  1 |
| Klinovszky 2019 (Hungry) | Cross sectional | 113 | 33.6% | 60.56 ± 12.9 | Diabetes | Statements about adherence-PE | - | Medication adherence B=-0.496, p<0.001 | 1 |
| Kim 2010 (Korea) | Cross sectional | 210 | 67.1% | 53.3 ± 10 | Metabolic syndrome | Regular exercise vs non regular exercise according to stages of change | - | HDL-C level OR=1.08, 95%CI=-2:-1.14, p=0.012; Consciousness raising OR=1.27, 95%CI=1.04-1.55, p=0.018;  Self-reevaluation OR=1.48, 95%CI=1.07-2.05, p=0.019;  Self-liberation OR=2.31, 95%CI=1.74-3.07, p=0.000 | 4  1  1  1 |
| Johnson 1998 (Australia) | Prospective longitudinal (cohort) | 251 | 73% | <60 years (34%) | AMI or angina | Self-report of adherence **(non-adherence)** | - | Perceived time barriers OR=3.67, 95%CI=1.17-11.5; Perceived physical environment barriers OR=3.79, 95%CI=1.31-11.0; Perceived benefits OR=0.27, 95%CI=0.09-0.81 | 1  1  1 |
| Huang 2015 (Taiwan) | RCT | 78 | 0% (all women) | 48.27 ± 8.03 | Breast cancer | Exercise diary | - | Fatigue OR=0.770, 95%CI=0.673-0.882, p=0.000; Interest in exercise OR=1.038, 95%CI1.006-1.072, p=0.023; Perceived importance of exercise OR=1.593, 95%CI=1.013-2.504, p=0.044; Employment status OR=6.103, 95%CI=1.037-35.925, p=0.046; Disease stage OR=0.426, 95%CI=0.230-0.788, p=0.008 | 4  1  1  2  4 |
| Heerema 2013 (Netherlands) | Cohort | 70 | 45.7% | 61.3 ± 10.2 | COPD | Maintenance program for 1 year | - | FEV_1_ OR=6.80, 95%CI=1.33-34.52, p=0.021; Depression OR=0.77, 95%CI=0.62-0.97, p=0.025;  Duration of rehabilitation OR=1.25, 95%CI=1.04-1.50, p=0.018 | 4  4  3 |
| Forechi 2018 (Brasil) | Cohort | 15.105 | 45.6% | <65 years (89.4%) | Dyslipidemia, hypertension, diabetes | Self-assessed PA time | - | Self-perceived health OR=0.40, 95%CI=0.31-0.53; Education OR=2.67, 95%CI=1.70-4.10; Income OR=1.96, 95%CI=1.50-2.57 | 1  2  2 |
| Fleury 2004 (US) | Cross sectional | 160 | 76% | 64 ± 10.2 | Heart failure | Maintenance to prescribed exercise program (Y/N) | Education X^2^=32.49, p=0.028;  Gender X^2^=6.80, p=0.009 | - | 2  2 |
| Farrokhzadi 2016 (Australia) | Cross sectional | 101 | 0% (all women) | 58.3 | Gynecological cancer | Active Australia Questionnaire | Pre-diagnosis PA OR=4.6, 95%CI1.1-18.5 | - | 1 |
| Duclos 2015 (France) | Cross sectional | 1766 | 63.5% | 63.5 ± 10 | Diabetes | MET scores | - | Being active OR=1.99, 95%CI=1.55-2.55, p<0.001;  Barriers to PA OR=1.30, 95%CI=1.10-1.54, p=0.002; Treatment burden OR=1.75, 95%CI=1.33-2.32, p<0.001;  Active physician OR=4.02, 95%CI=1.98-8.18, p<0.001 | 1  1  3  5 |
| Dohnke 2010 (Germany) | Cohort | 456 | 90% | 57.69 ± 10.03 | Heart failure | Frequency measure **(non-adherence)** | - | Intention OR=0.30, 95%CI=0.10-0.95, p<0.01;  Self-efficacy OR=0.60, 95%CI=0.37-0.95, p<0.05 | 1  1 |
| D'Andrea 2014 (US) | Pooled 1997–2010 National Health Interview Survey data | 2378 | 55.1% | <65 years (32%) | Colorectal cancer | Frequency and intensity of LTPA **(non-adherence)** | - | >2 chronic conditions OR=0.40, 95%CI=0.26-0.61, p=0.04;  Current smoker OR=0.49, 95%CI=0.31-0.79; Education OR=1.77, 95%CI=1.19-2.64 | 4  2  2 |
| Courneya 2012 (Canada) | RCT | 122 | 59% | 53 | Lymphoma | GLTEQ | - | Intention B=0.18, p=0.034; Accepting a post-intervention exercise prescription B=0.33, p=0.001; PBC B=0.18, p=0.028 | 1  1  1 |
| Courneya 2002 (Canada) | RCT | 96 | 16.6% | 51.55 ± 10.15 | Cancer | LSI or GLTEQ | - | Past exercise B=0.36, p<0.001; Sex B=0.30, p<0.001;  Intention B=0.14, p=0.080 | 1  2  1 |
| Courneya 2009 (Canada) | RCT | 201 | 0% (all women) | <50 years (56.7%) | Breast cancer | GLTEQ) | - | Pretrial exercise B=0.23, p=0.002; Age B=-0.15, p=0.028;  Breast conserving surgery B=0.15, p=0.033; instrumental attitude B=0.14, p=0.045 | 1  2  3  1 |
| Courneya 2004 (Canada) | RCT | 93 | 58.1% | 60.3 ± 10.4 | Colon cancer | LSI or GLTEQ | - | Exercise stage B=0.35, p=0.001; Employment status B=-0.28, p=0.010; Treatment protocol B=-0.26, p=0.018; PBC B=0.20, p=0.055 | 1  2  3  1 |
| Corvera 2004 (US) | Secondary analysis of RCT | 39 | 100% | 63.4 ± 10.4 | Heart failure | Pedometer for minutes of walking time **(non-adherence)** | - | Comorbidity OR=2.7, 95%CI=1.11-6.71, p=0.03;  HF duration OR=1.1, 95%CI=1.01-1.13; BMI OR=0.76, 95%CI=0.58-0.98; Negative emotion OR=0.47, 95%CI=0.24-0.91 | 4  4  4  4 |
| Chlebowy 2016 (US) | Secondary analysis of RCT | 26 | 34.6% | 55.8 ± 2.1 | Diabetes | Self-assessed PA | - | Age OR=2.84, 95%CI=1.48-10.81, p=0.014;  Education OR=3.12, 95%CI=1.93-7.46, p<0.001 | 2  2 |
| Chipperfield 2013 (Australia) | Cross sectional | 365 | 100% | 67.4 ±7.5 | Prostate cancer | IPAQ | - | Depression OR=0.84, 95%CI=0.76-0.94, p<0.01;  Education OR=0.61, 95%CI=0.38-0.97, p<0.01 | 4  2 |
| Blanchard 2009 (Canada) | Cohort | 76 | 76% | 62.64 ± 10.98 | Coronary heart disease | GLTEQ or LSI | Intention zero-correlation 0.31, p<0.001, B=0.23; Self-efficacy zero-correlation 0.37, p<0.001, B=0.32 | - | 1  1 |
| Blanchard 2008 (Canada) | Cohort | 76 | 76% | 62.64 ± 10.98 | Heart disease | GLTEQ or LSI | Attitude zero-correlation 0.41, p<0.001;  PBC zero-correlation 0.40, p<0.001;  Intention zero-correlation 0.31, p<0.001; Implementation intention zero-correlation 0.39, p<0.001 | - | 1  1  1  1 |
| Basen 2013 (US) | Cohort | 100 | 0% (all women) | 57.0 ± 11.01 | Endometrial cancer | Minutes of exercise completed per day | - | Self-efficacy F(1, 2254)=215.82, p<0.0001 | 1 |
| Baima 2017 (US) | Single arm prospective trial | 14 | 46.7% | 55 ± 3.4 | Brain tumor | Frequency measure | Being married p=0.033;  Income p=0.047; Physical well-being p=0.047; Social well-being p=0.018 | - | 2  2  2  2 |
| Ali 2017 (Pakistan) | Cross sectional | 265 | 59.2% | 67.2 ± 13.4 | Coronary heart disease + CABG **(non-adherence)** | Frequency measure | Reluctant to follow exercise regimen OR= 14, 95%CI-6.3-31.4, p<0.001; Too busy OR=9.2, 95%CI=5.2-16.4, p<0.001;  Fear it will aggravate heart issues OR=2.7, 95%CI=1.5-5, p<0.001;  Feel fatigued after exercising OR=2.6, 95%CI=1.5-5, p=0.005 | - | 1  1  4  4 |
| Mi-wha 2019 (Korea) | Cross sectional | 282 | 60.6% | 62.27 ± 9.30 | Coronary heart disease | International Physical Activity Questionnaire (IPAQ) |  | Motivation B=0.31, p=0.002;  Social support B=0.50, p=0.002;  Competence B=0.27, p=0.002 | 1  2  1 |
| Hyoung-Sook 2020 (Korea) | Cross sectional | 152 | 0% | 49.48 ± 9.47 | Breast cancer | Establishment and commitment to an exercise plan |  | Income B=0.22, p=0.001;  Exercise history B=0.20, p<0.001;  Current exercise B=0.68, p<0.001 | 2  1  1 |
| Caetano 2020 (Brazil) | Cross sectional | 93 | 45% | 62 ± 12 | Chronic stroke | First question of the first section of the EPQ(stroke)-Brazil |  | Self-efficacy OR=7, 95%CI=2.8-17.5, p = 0.0001;  Walking speed OR=56, 95%CI=5-617, p = 0.001 | 1  4 |
| Stone 2019 (Canada) | RCT | 400 | 0% | 59.4 ± 5.0 | Breast cancer | Exercise journals and Polar FT4 heart rate monitor |  | BMI B= -0.77, p = 0.01;  Employment status B= -6.65, p = 0.02;  Physical health B= 0.51, p = 0.03 | 4  2  2 |

Domain: 1 = Patient-related, 2 = Social/economic, 3 = Therapy-related, 4 = Condition-related, and 5 = Health system factors

# **Appendix 6: Covariates within multivariable and adjusted models**

| **Author (year)** | **Multivariable or adjusted model** | **Covariates** |
| --- | --- | --- |
| Parker (2021) | Adjusted model | Motivation, Self-efficacy, Tumor type, Evidence of disease at the time of the survey, BMI, Chemotherapy within 6 months of the survey |
| Leung (2020) | Adjusted model | Self-efficacy, Stage of Change, Current drinking habit, Fulltime work, Jointed CNSLMP before, Had PA consultation |
| Cheng (2019) | Multivariable model | Social support, Age,  Gender, Exercise history, Program comprehension, Patient  motivation |
| Albert (2012) | Adjusted model | Perception of good health, Self-confidence for exercise, Lower barriers, Attitude, Perceived value, Benefits, Knowledge, BMI, Gender, Smoking status, Insurance type |
| Acar (2015) | Adjusted model | Education, Regular follow-up, Age, Comorbid conditions, Revascularization type |
| Woodgate (2005) | Multivariable model | Self-efficacy, Past exercise |
| Williams (2002) | Adjusted model | Self-efficacy, Social support |
| Tovar (2007) | Adjusted model | Susceptibility, Severity, Benefits, Barriers, Self-efficacy |
| Tooth (1993) | Multivariable model | Occupation, Expectation, Age, Exercise knowledge, Previous weekly met hours of activity |
| Wilson (1986) |  |  |
| Mosleh (2015) | Multivariable model | Age, BMI, Gender, Marital status, Medical comorbidity, Health education |
| Miller (1990) | Multivariable model | Attitude, Perceived believes of others, Intention |
| McGuire (2011) | Multivariable model | Previous adherence, Marital status, BMI, Comorbidities, Support, Knowledge and Skills, Self-efficacy, Feedback |
| Luszczynska (2006) | Multivariable model | Physical activity before MI, Nutrition before MI, Intention, Perceived support, Having partner or spouse |
| Olson (2014) | Multivariable model | Fatigue, Self-efficacy, Social support, Facilitators, Barriers |
| Ng (2021) | Adjusted model | Gender, Well-being, Barriers, Enjoyment, Higher functional score, New or Follow-up patient, Type of physician |
| Murray (2012) | Multivariable model | Self-efficacy, Age, Gender, Exercise capacity, Income, Education, Social status, Perceived control |
| Moore (2003) | Multivariable model | Age, Comorbidity, Social support, Benefits/Barriers |
| Kim (2010) | Multivariable model | Smoking, Blood pressure, HDL cholesterol, Consciousness raising, Dramatic relief, Self-reevaluation, Social liberation, Counter conditioning, Self-liberation, Stimulus control, Pros, Cons, Self-efficacy |
| Johnson (1998) | Multivariable model | Enjoyment, Time, Health, Physical environment, Social environment, Barriers, Benefits |
| Huang (2015) | Multivariable model | Fatigue, Interest in exercise, Perceived importance of exercise, Employment status, Disease stage |
| Heerema (2013) | Multivariable model | FEV_1_, Total lung capacity, Depression, Duration of rehabilitation |
| Forechi (2018) | Adjusted model | Age, Race, Education, Income, Self-perceived health, Smoking status, Alcohol use, Morbidity score, BMI, Opportunity to practice physical activity, Conditions to practice physical activity |
| Duclos (2015) | Multivariable model | Barriers, Being active, Number of antidiabetics, Active physician |
| Dohnke (2010) | Multivariable model | Intention, Self-efficacy, Gender |
| D’Andrea (2014) | Adjusted model | Age, Gender, Race, Marital status, Education, Poverty, Health insurance, BMI, ≥ 2 chronic conditions, Smoking status, Alcohol status, Survey year, Time since diagnosis |
| Courneya (2012) | Multivariable model | Accepting a  post-intervention exercise prescription, Peak power output, PBC, Having Hodgkin lymphoma, Intention |
| Courneya (2002) | Multivariable model | Experimental condition, Past exercise, Sex, Intention, Attitude, Treadmill time, PBC, Control beliefs |
| Courneya (2009) | Multivariable model | Pretrial exercise, Age, Surgery strategy, Strength, Postintervention fatigue, Attitude, BMI |
| Courneya (2004) | Multivariable model | Intention, PBC, Exercise stage, Tumor stage, Chemotherapy, Radiotherapy, Treatment protocol, Age, Employment status |
| Corvera (2004) | Multivariable model | BMI, Comorbidity score, Peak VO_2_, HF duration, Emotional dysphoria |
| Chlebowy (2016) | Multivariable model | Gender, Age, Education |
| Chipperfield (2013) | Multivariable model | Education, Comorbid conditions, Depression, Anxiety, FACT-P PCS score, Age, Treatment |
| Basen (2013) | Multivariable model | Baseline exercise, Self-efficacy, Outcome expectations, BMI, Education, |

# **Appendix 7: Risks of bias traffic light plots per domain**

**Patient-related**


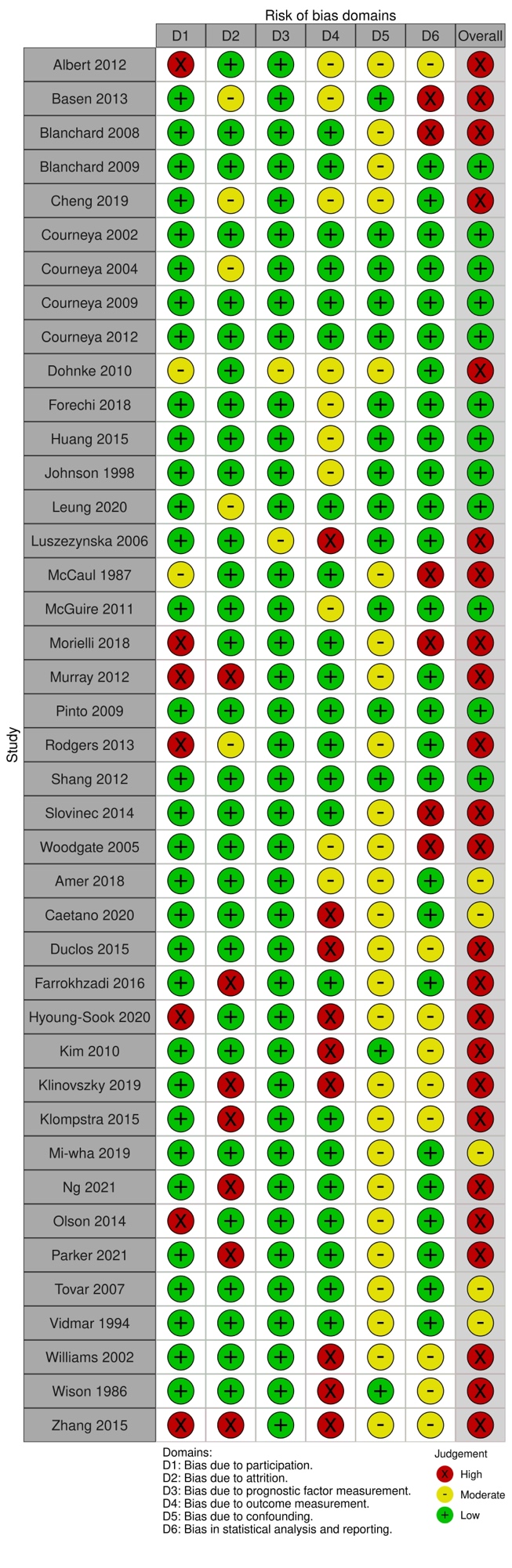


**Social-economic**

**
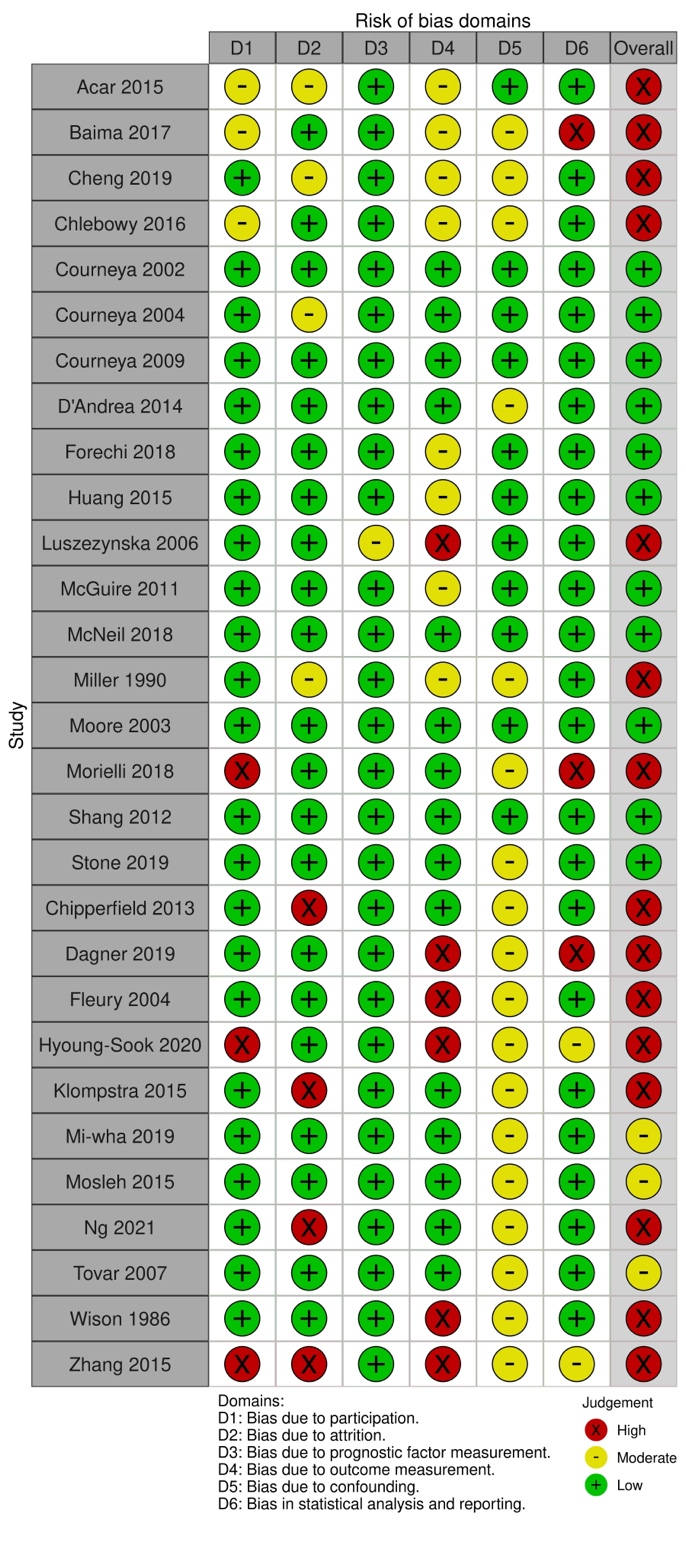
**

**Therapy-related**

**
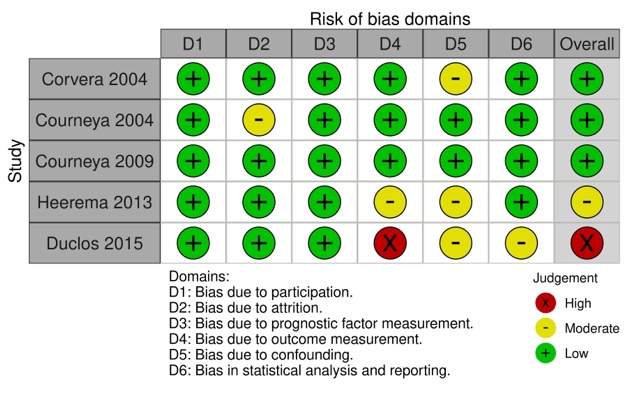
**

**Condition-related**

**
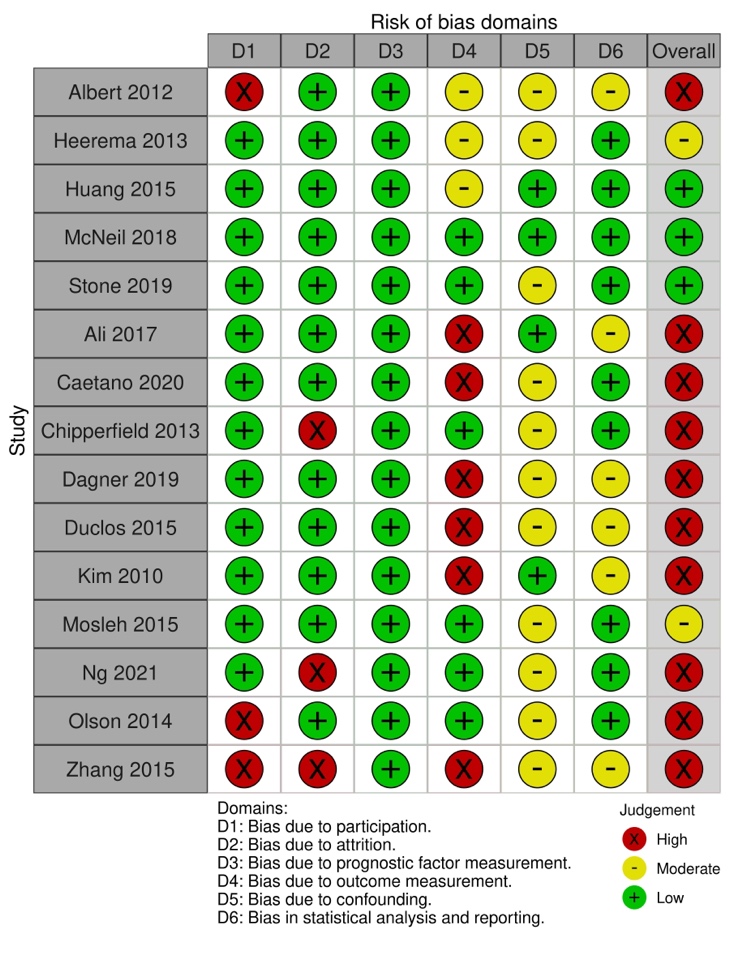
**

**Health-system-related QUIPS**

**
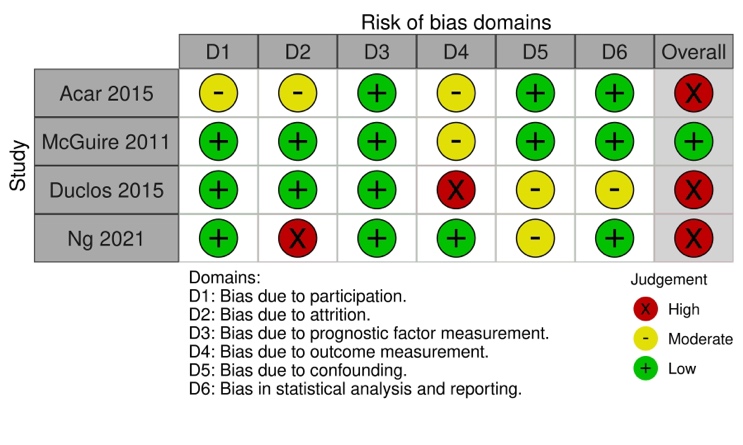
**

# **Appendix 8: Publication bias funnel plots**

**Self-efficacy**


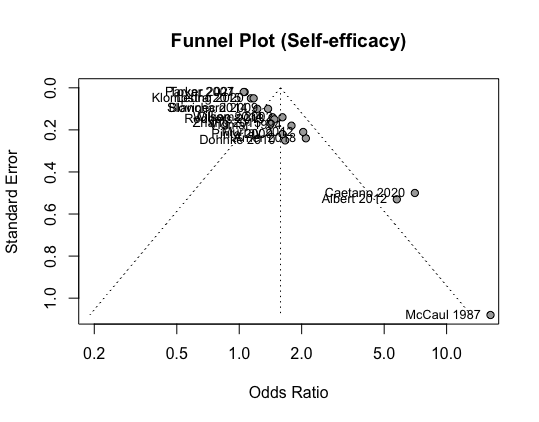


**Egger’s test**

Test result: t = 16.47, df = 16, p-value < 0.0001

**Exercise history**


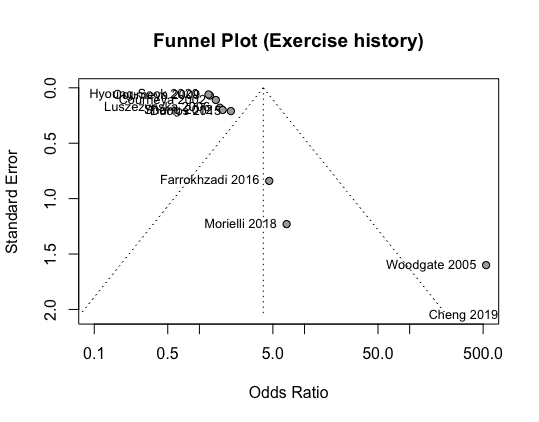


**Egger’s test**

Test result: t = 7.59, df = 8, p-value < 0.0001

# **Appendix 9: Forest plots per domain**

**Patient-related**

**Self-efficacy**


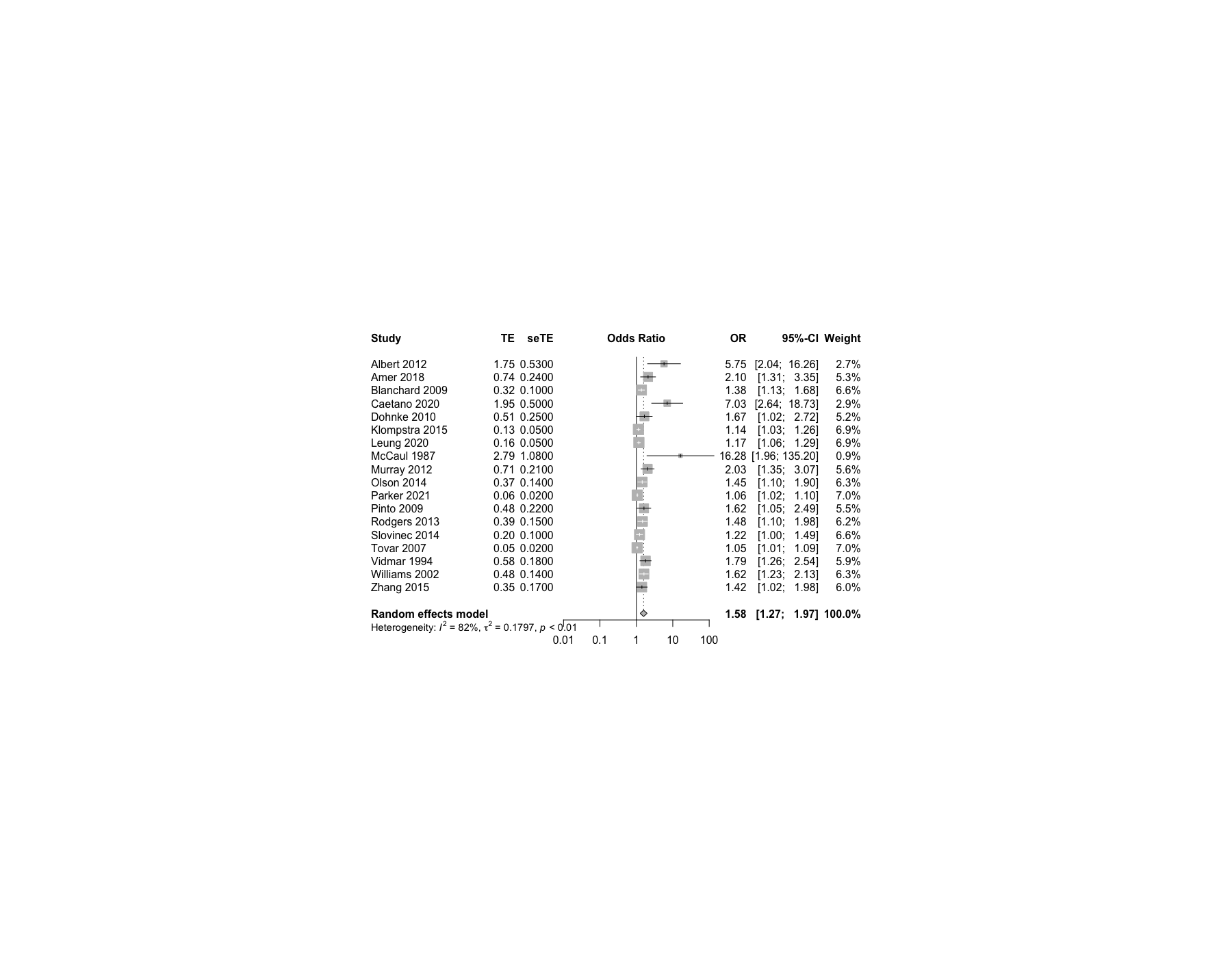


**Exercise history**

**
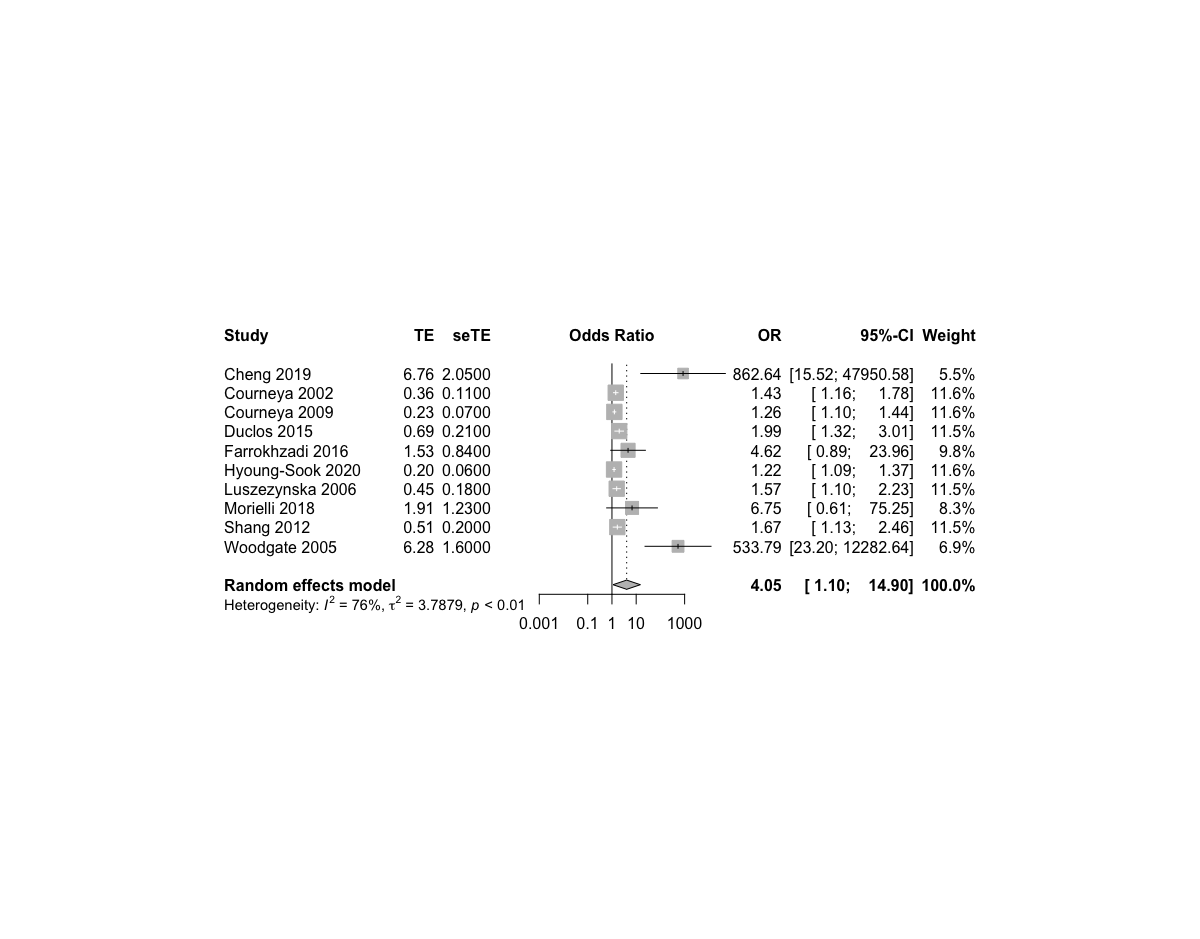
**

**Intention**

**
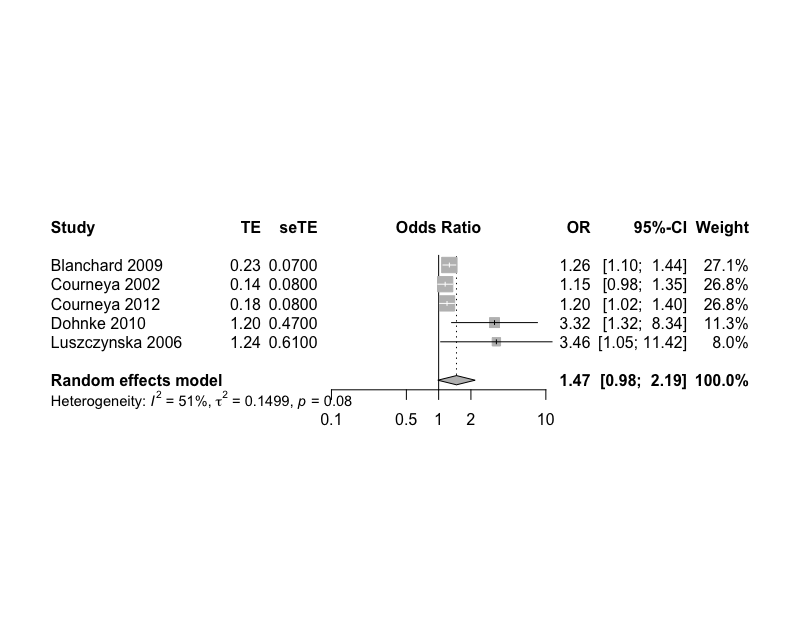
**

**Motivation**

**
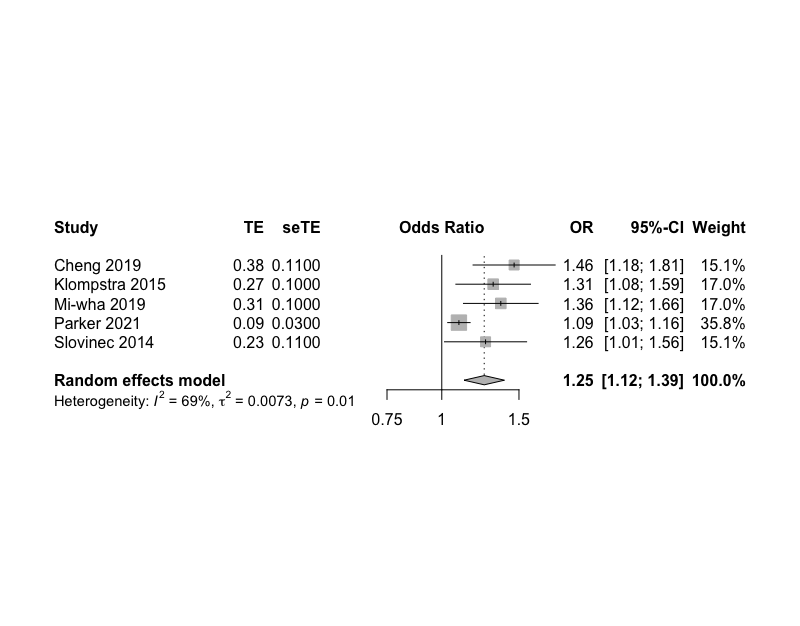
**

**Attitude**

**
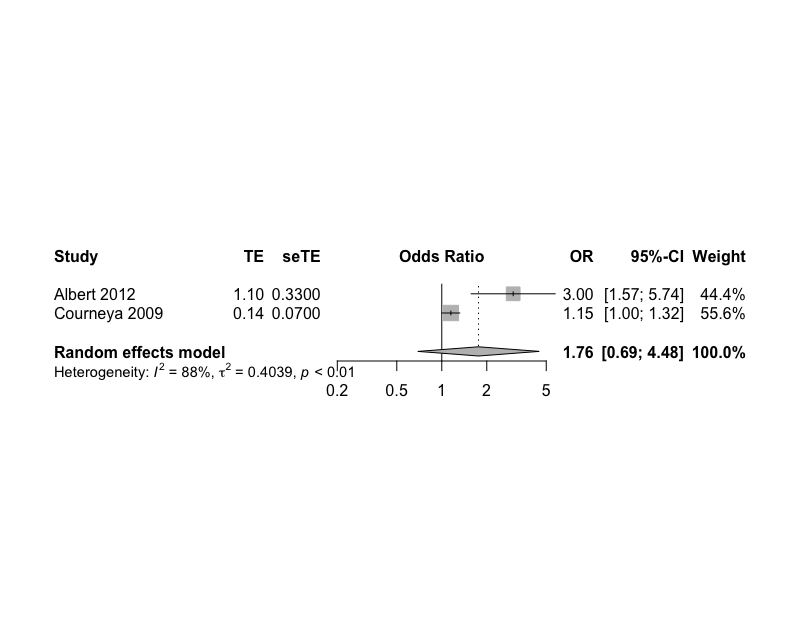
**

**PBC**

**
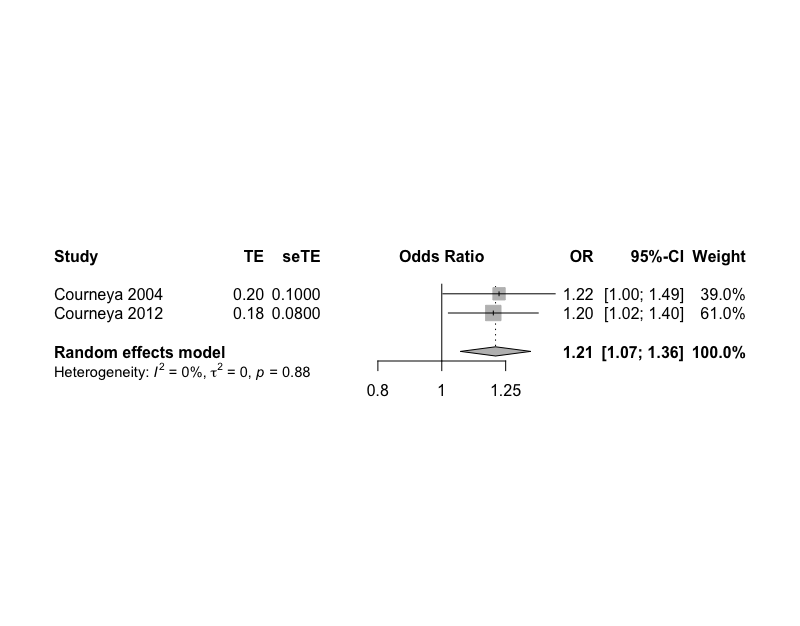
**

**Perceived benefits**

**
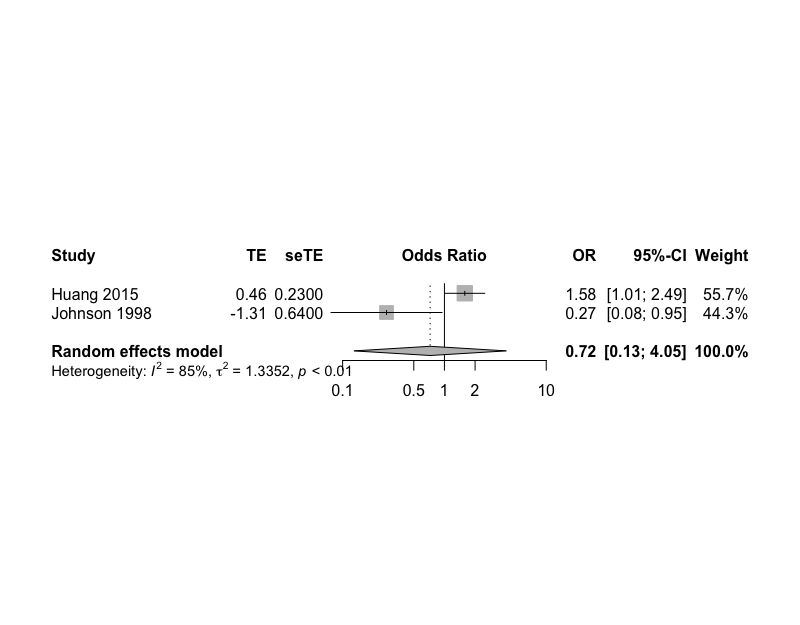
**

**Social-economic**

**Education**


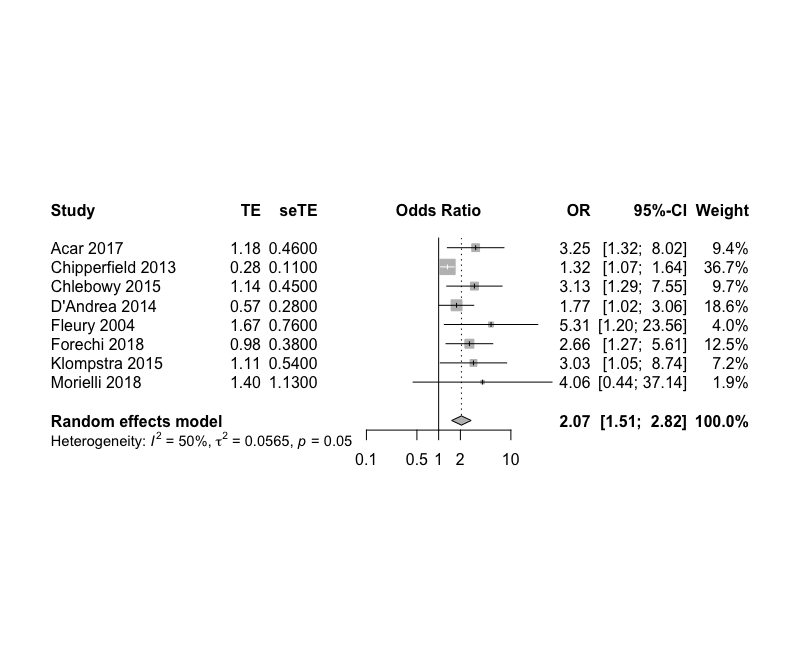


**Social support**


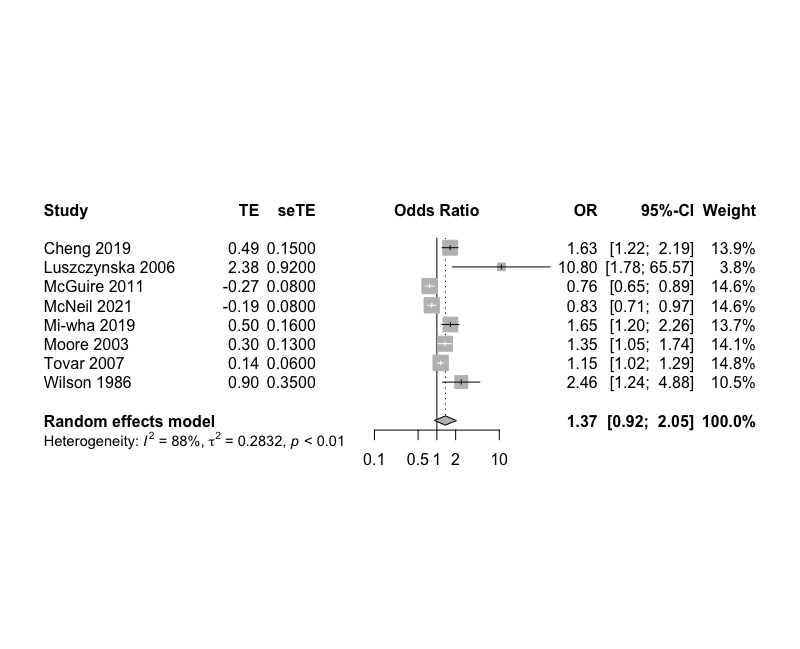


**Age**


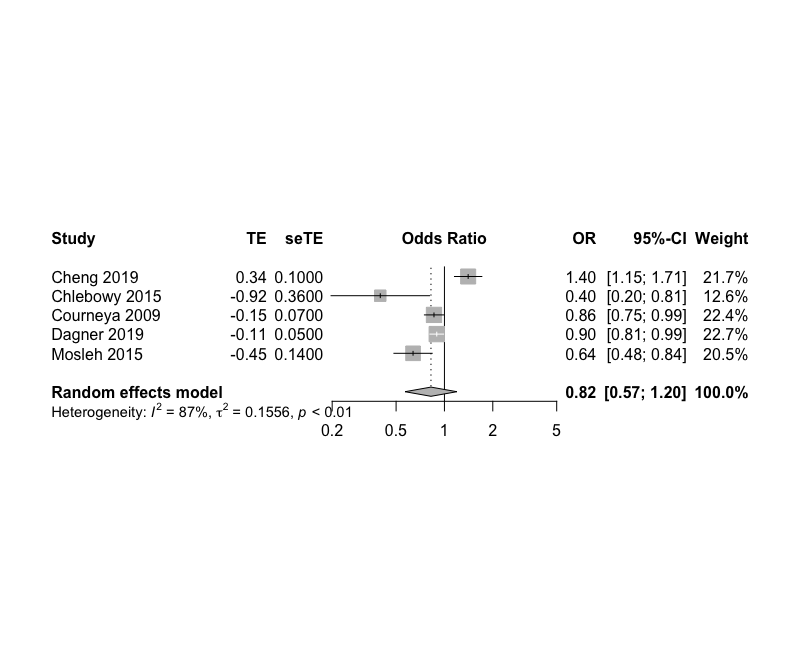


**Gender**


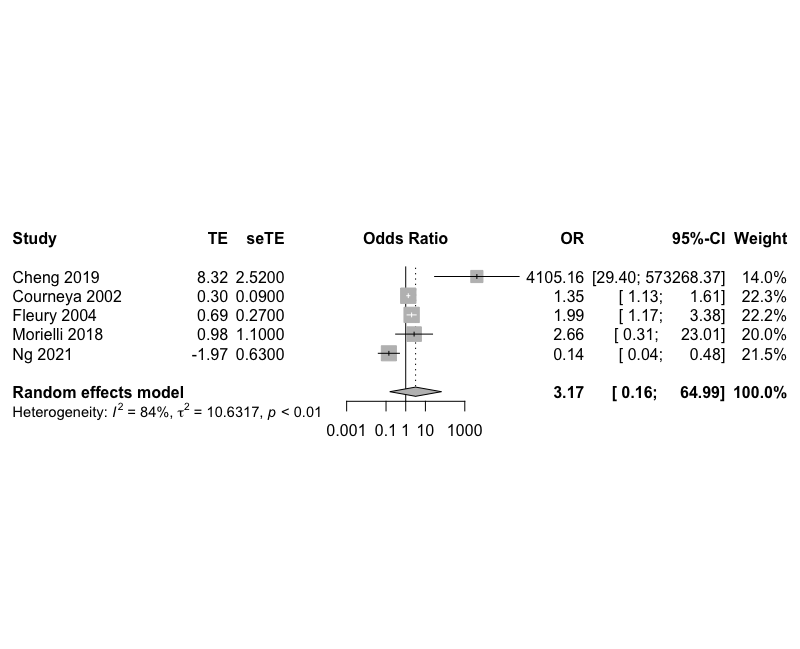


**Employment status**


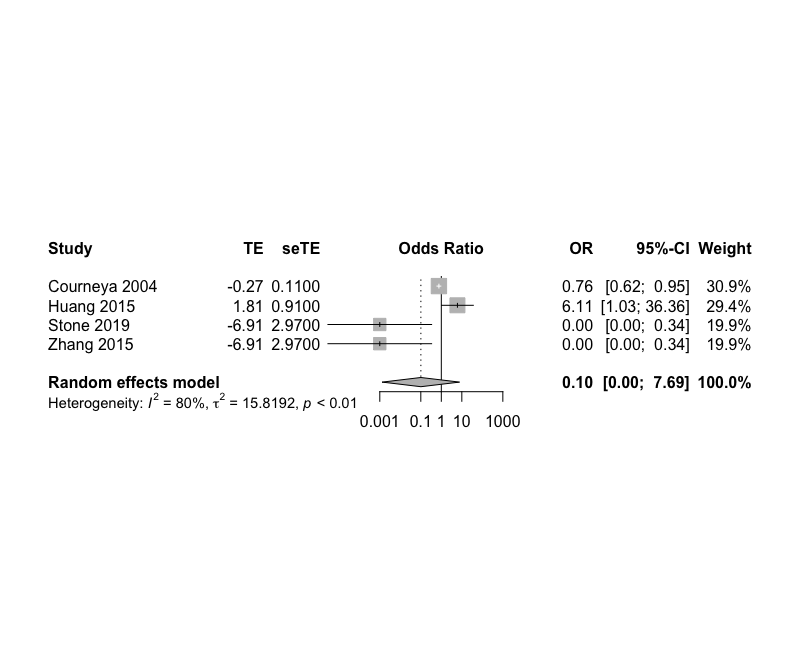


**Income**


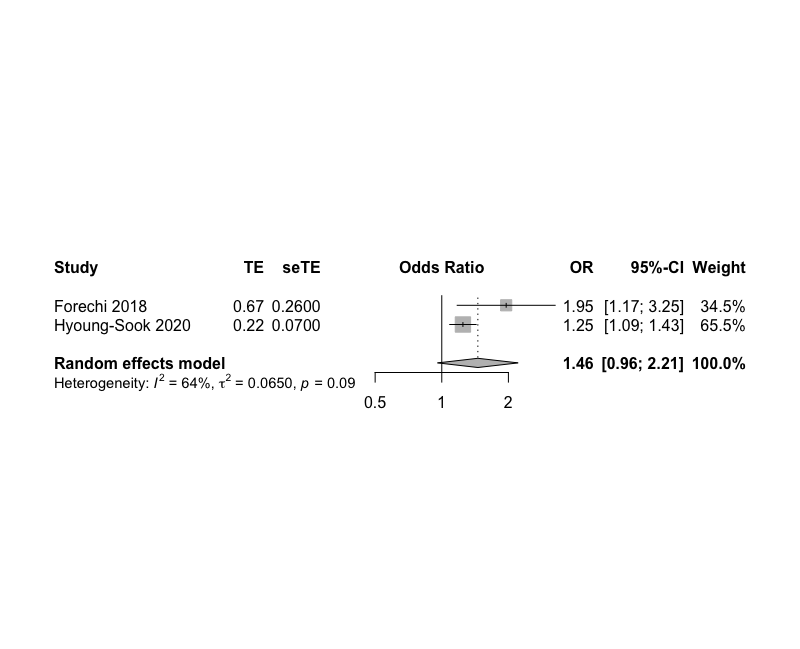


**Marital status**


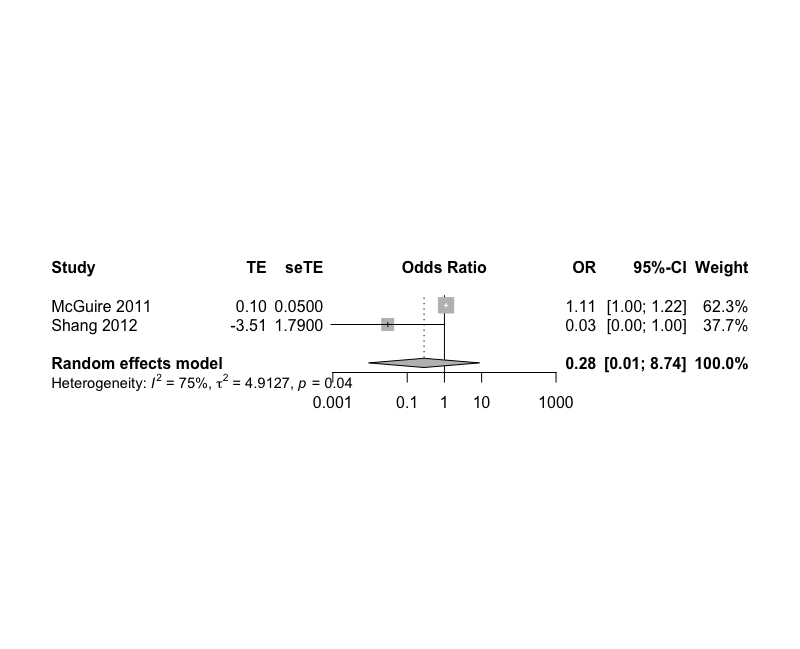


**Physical Health**


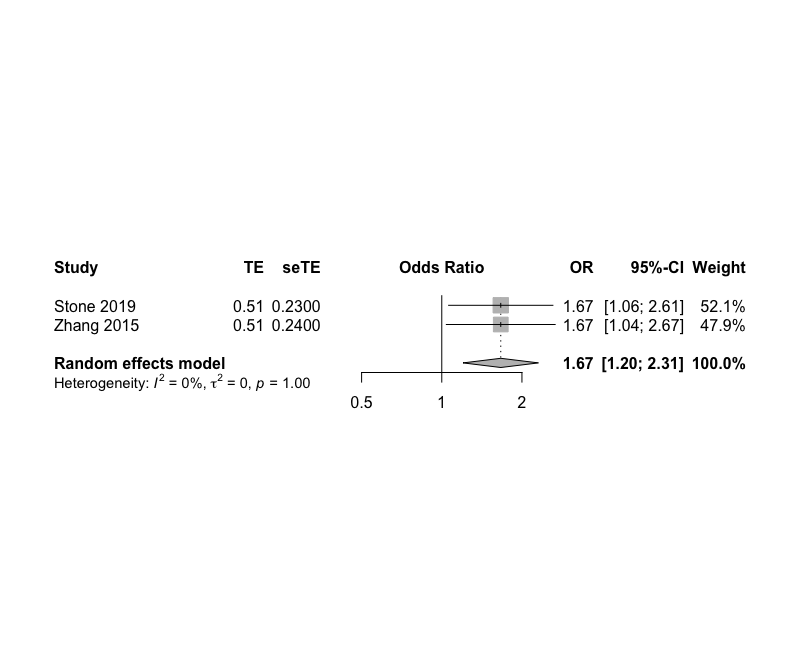


**Therapy-related**


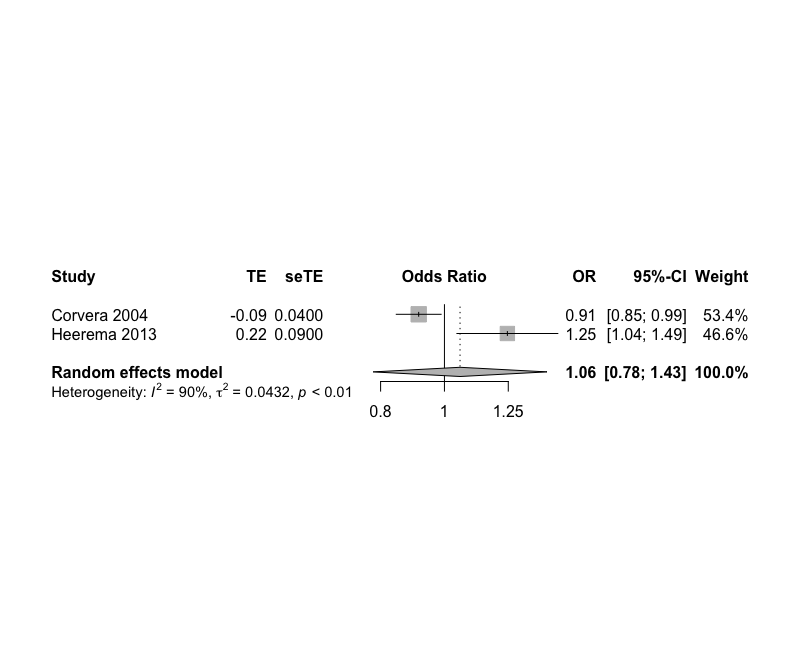


**Condition-Related**

**BMI**


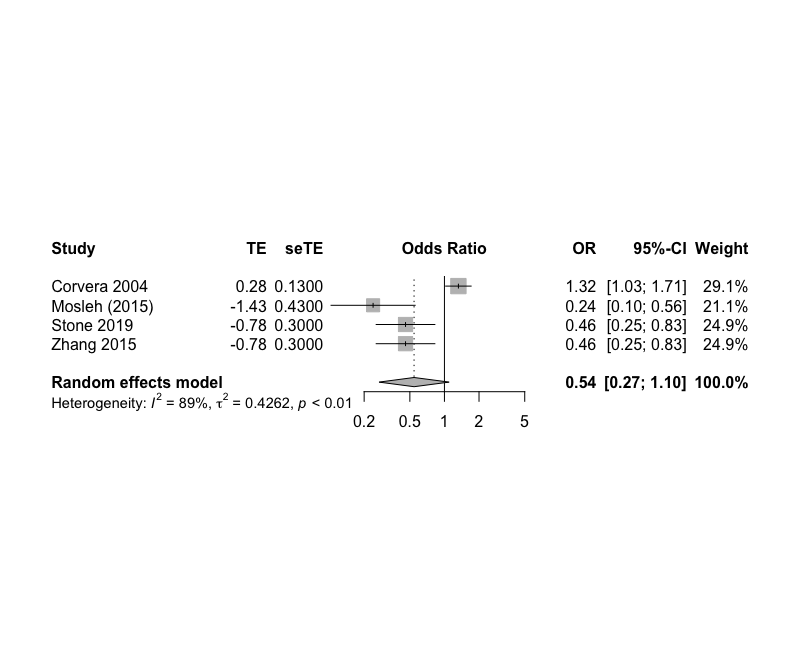


**Comorbidity**


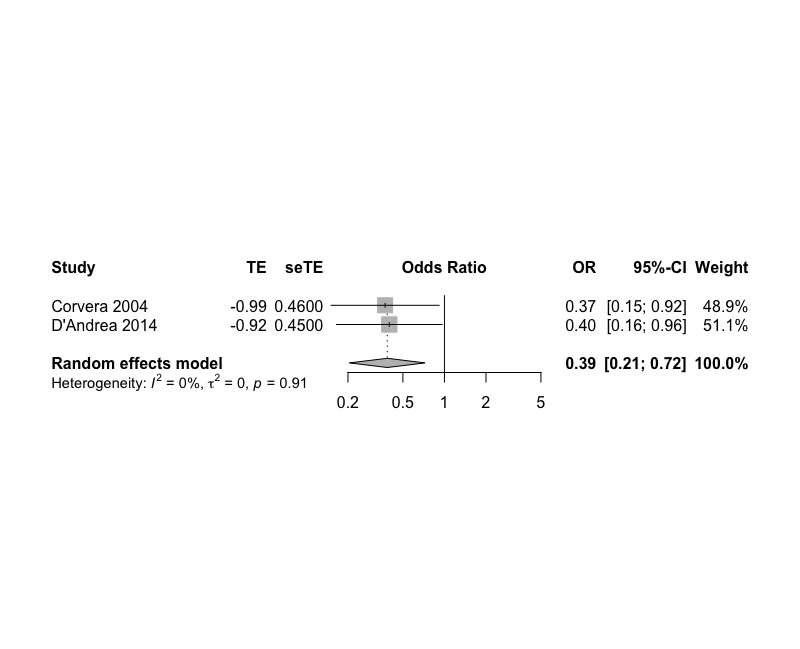


**Depression**


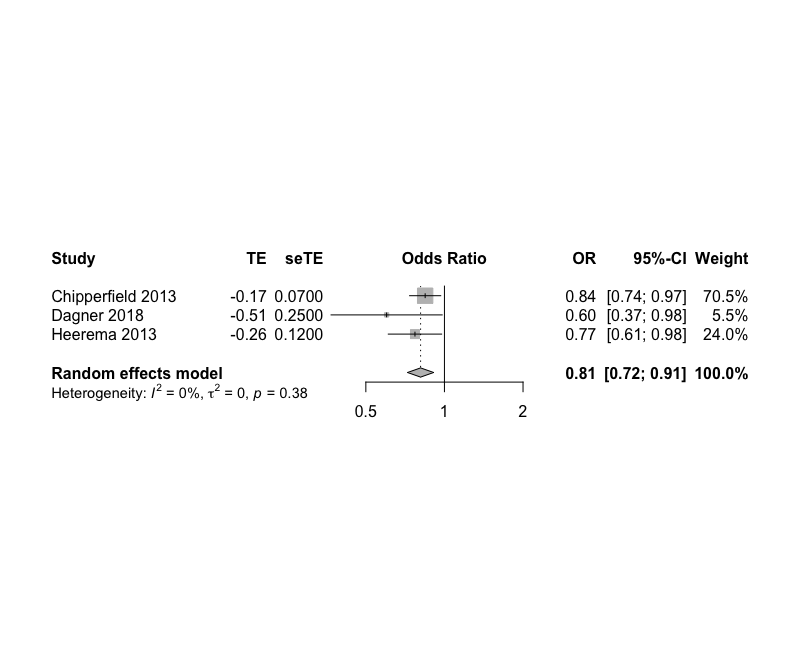


**Fatigue**


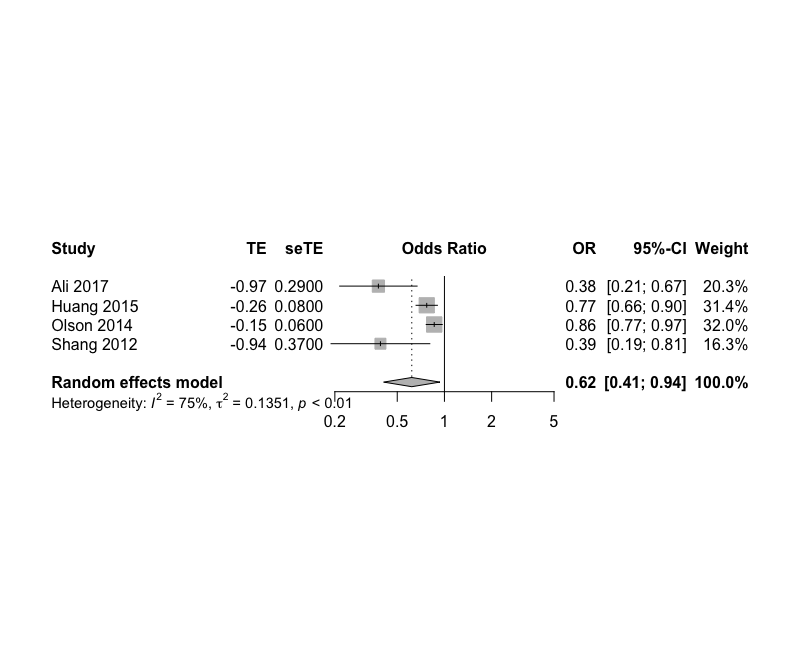


# **Appendix 10: Subgroup- and sensitivity analysis**

**Self-efficacy**

**Subgroup on disease**


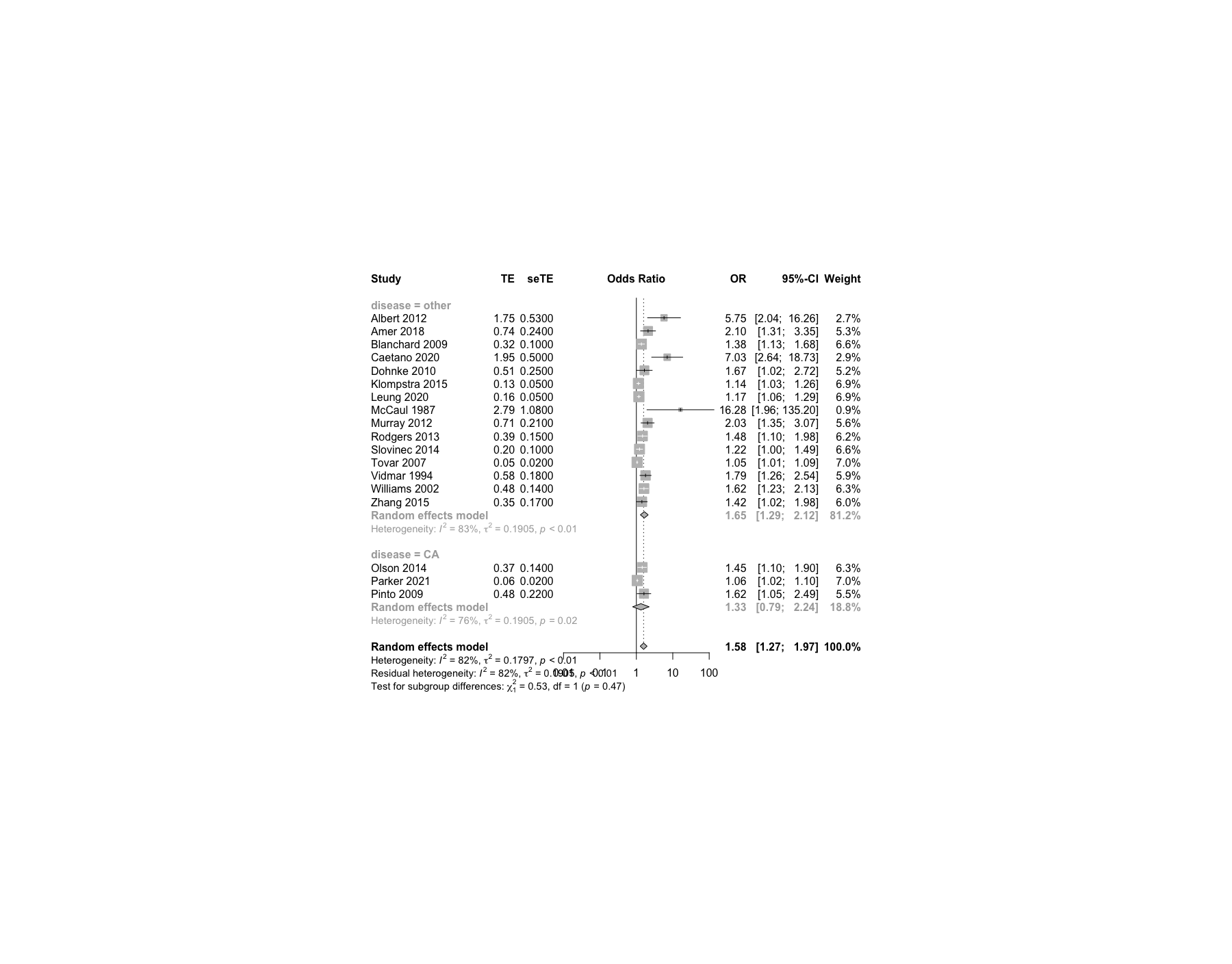


**Subgroup on design**

**
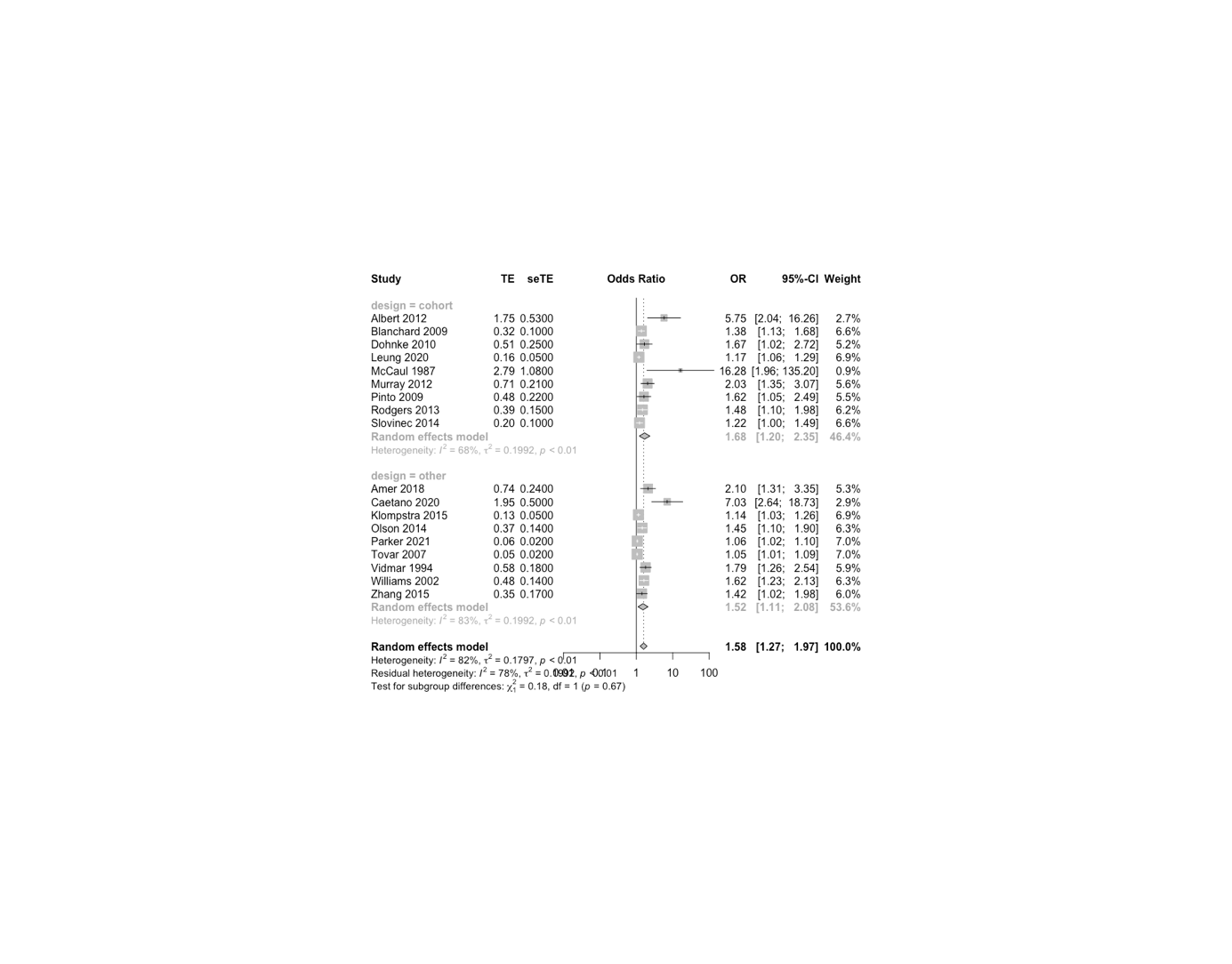
**

**Sensitivity analysis**

Leaving the studies of Albert, McCaul and Caetano out of the analysis


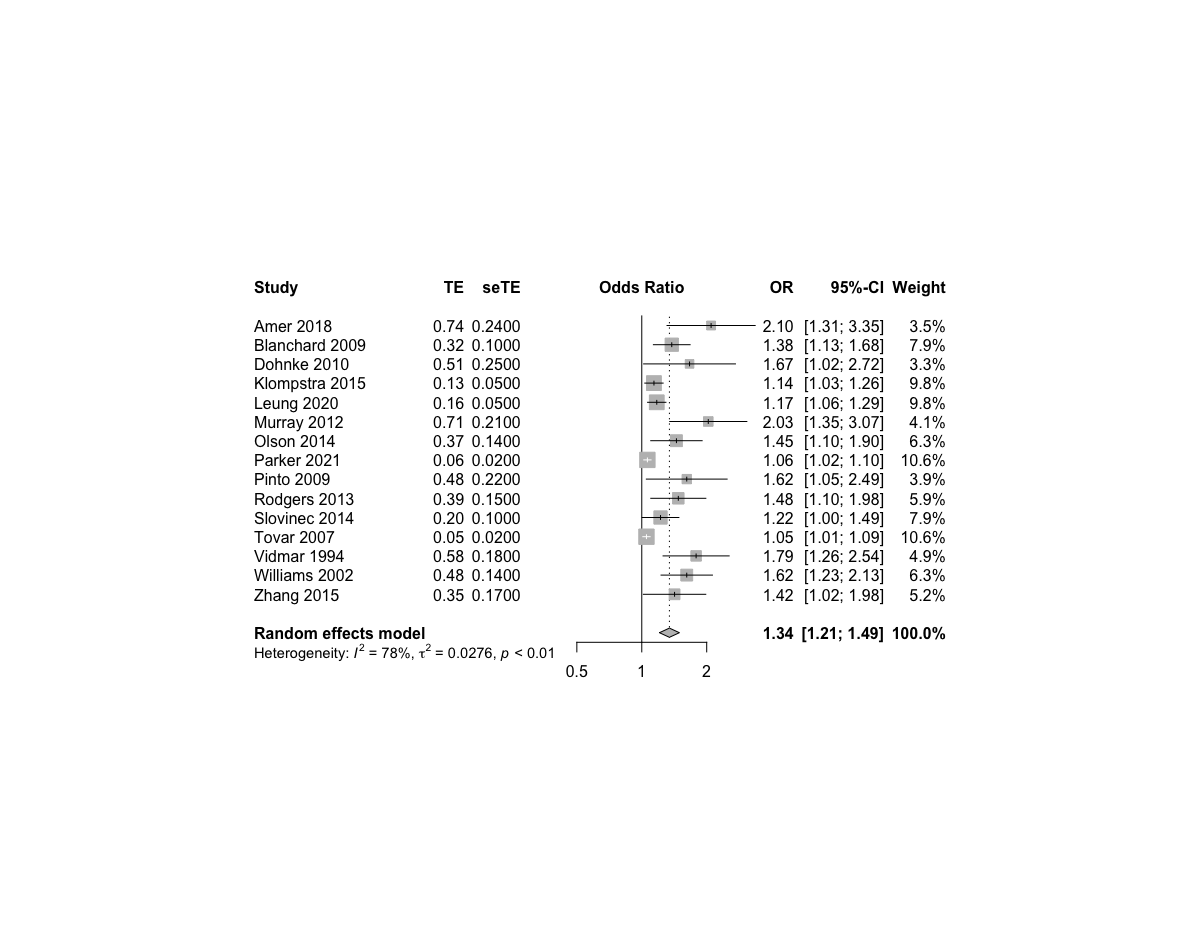


**Subgroup on disease**


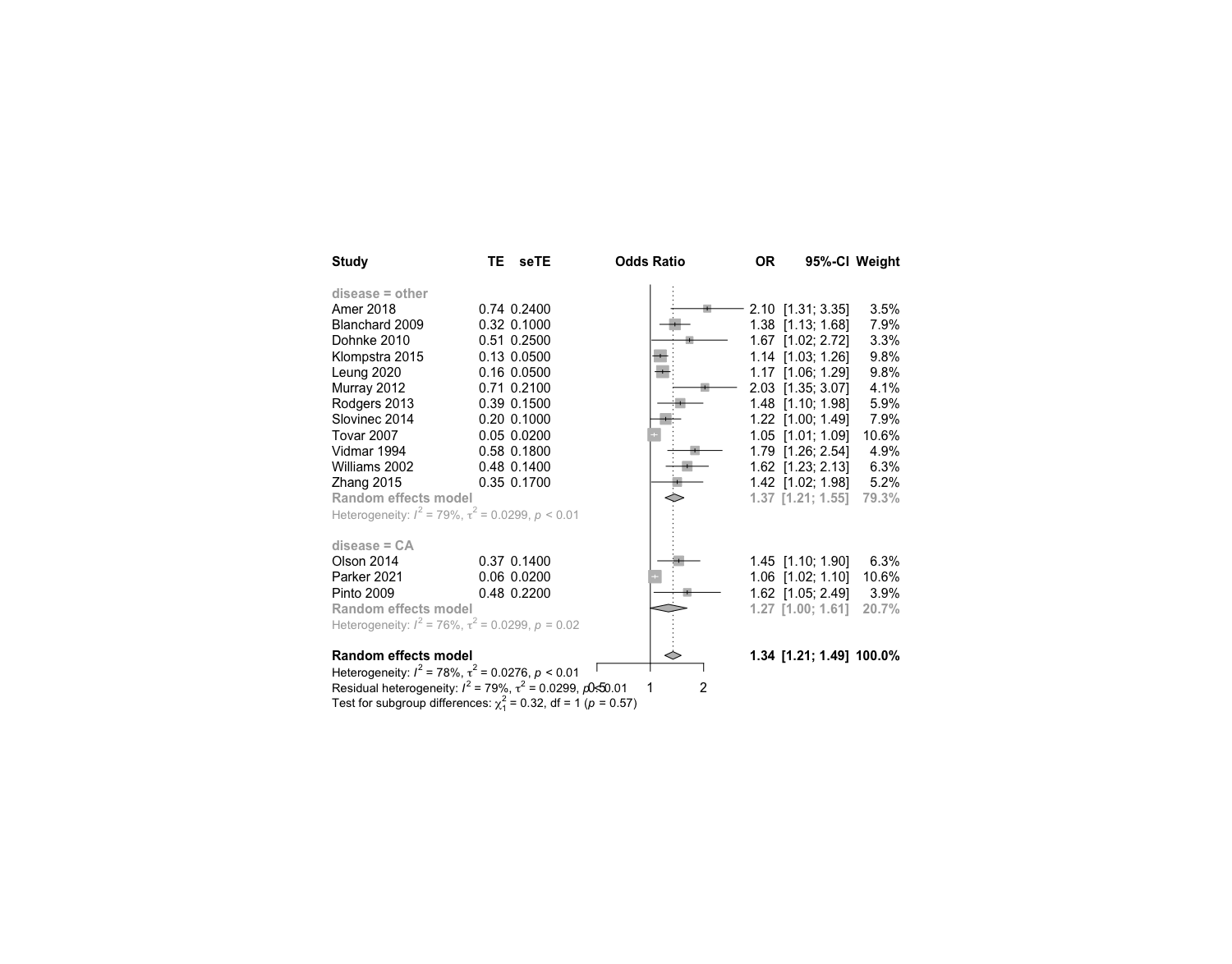


**Subgroup on design**


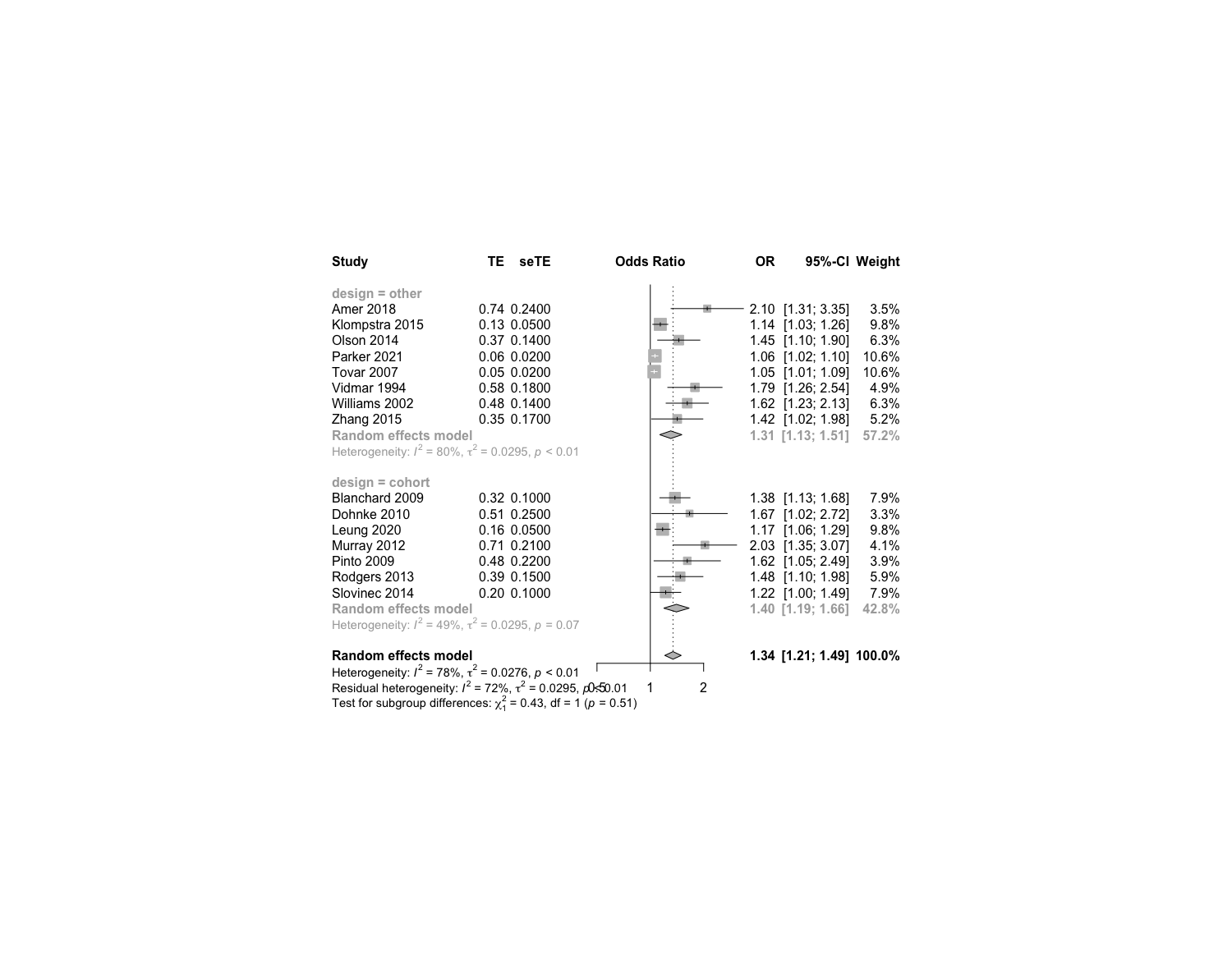


**Exercise history**

**Subgroup on disease**

**
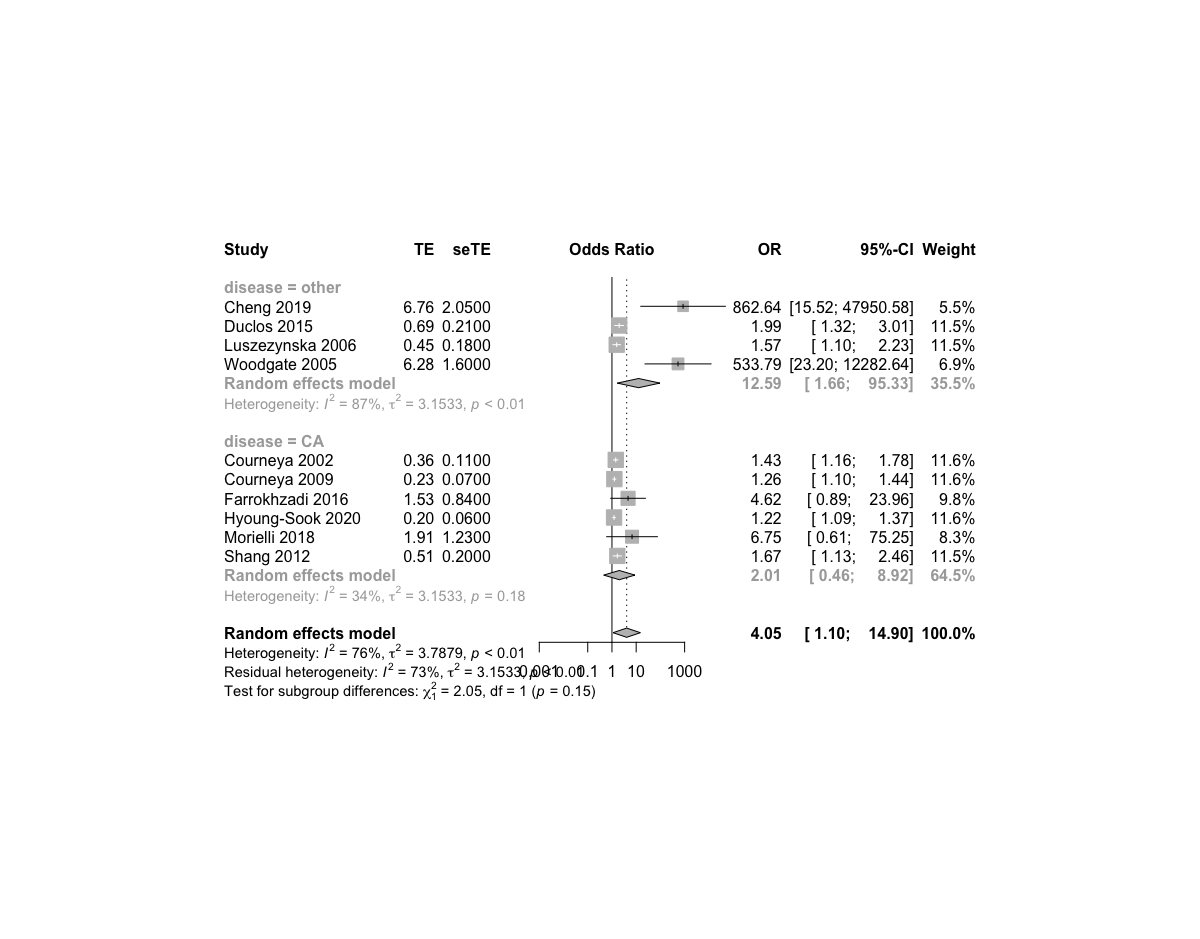
**

**Subgroup on design**


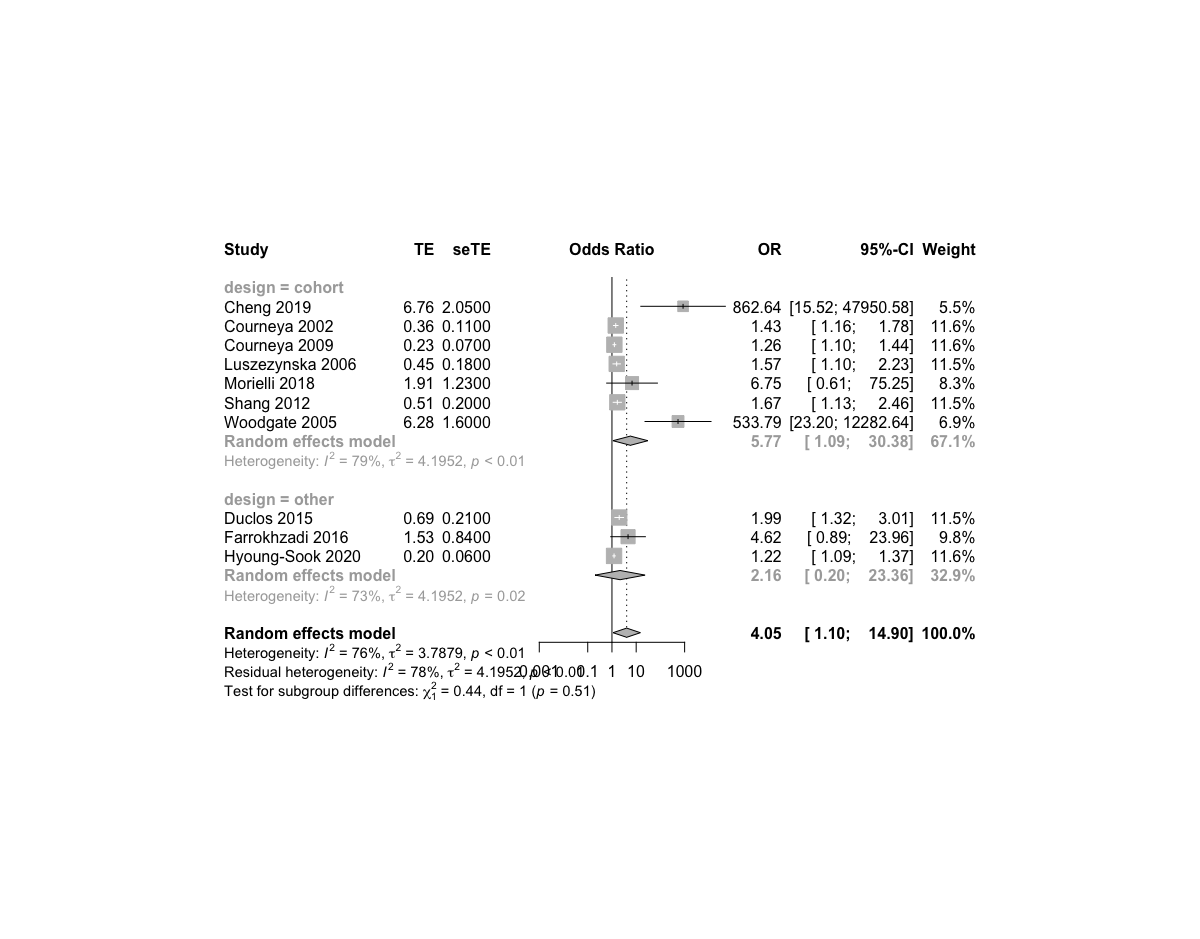


**Sensitivity analysis**

Leaving the studies of Cheng and Woodgate out of the analysis

**Exercise history**

**
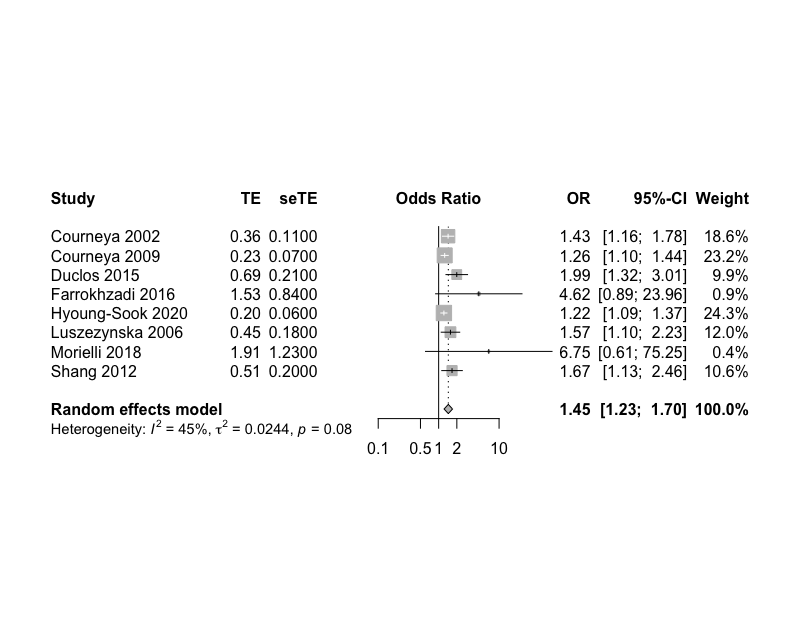
**

**Subgroup on disease**

**
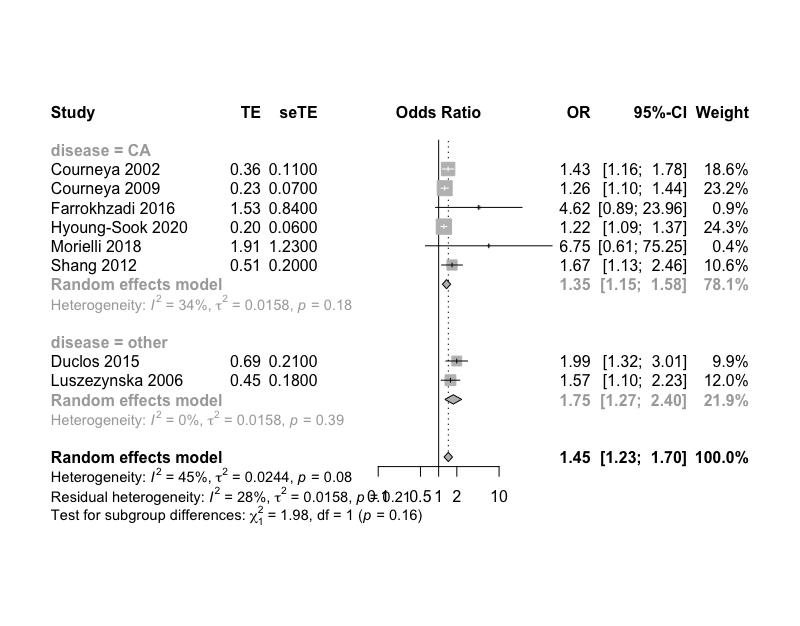
**

**Subgroup on design**

**
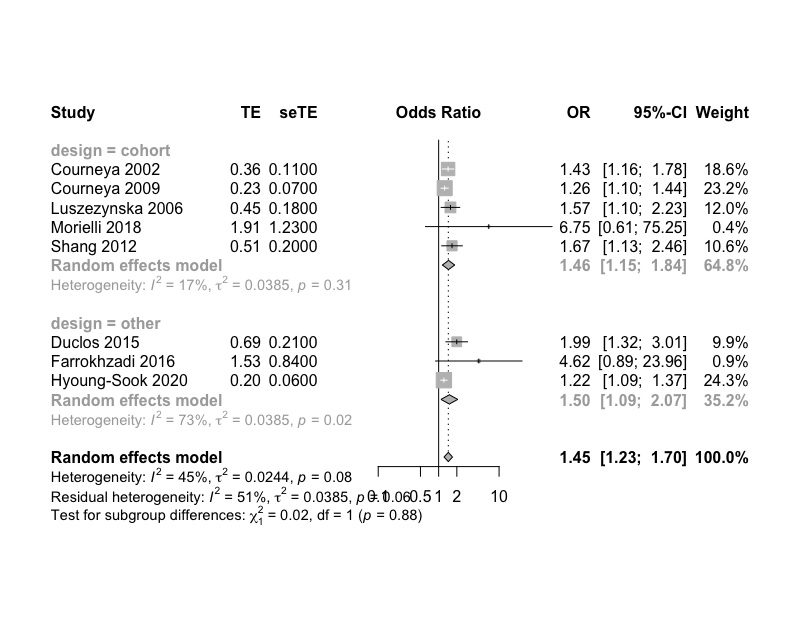
**

**Motivation**

**Subgroup on disease**

**
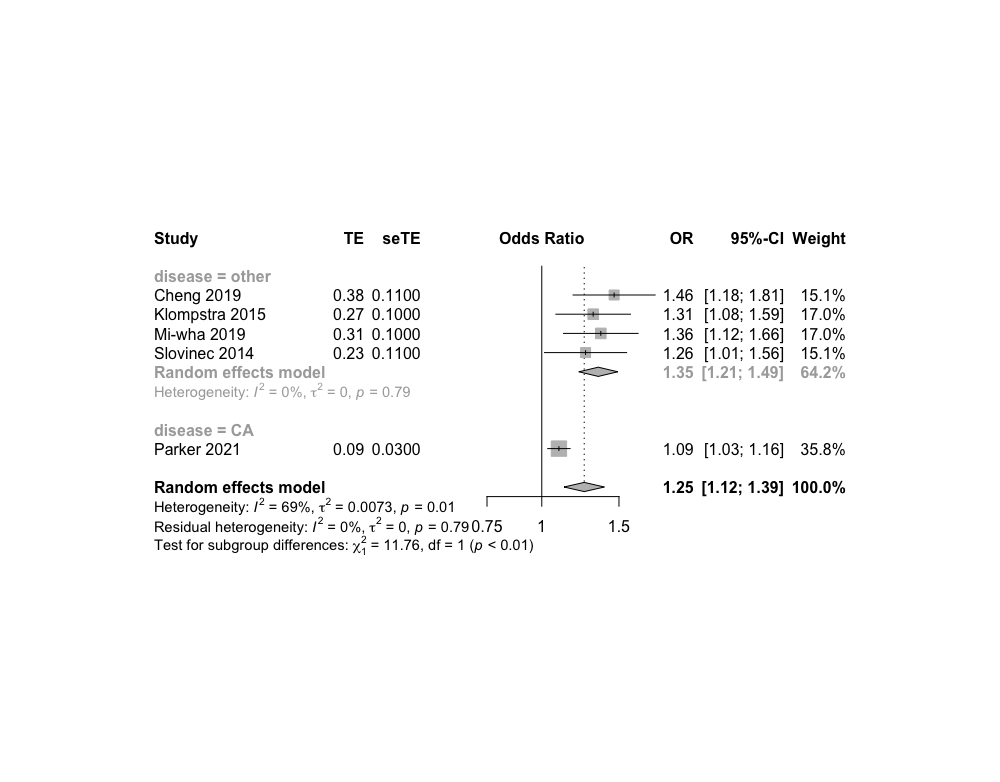
**

**Subgroup on design**

**
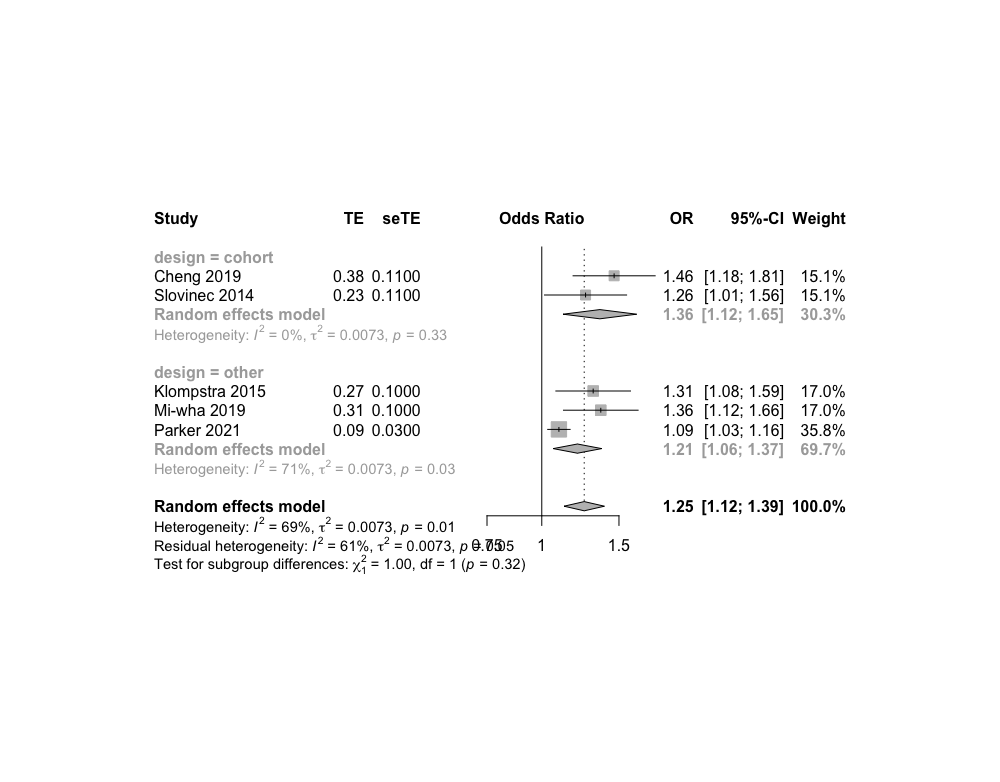
**

**Education**

**Subgroup on disease**

**
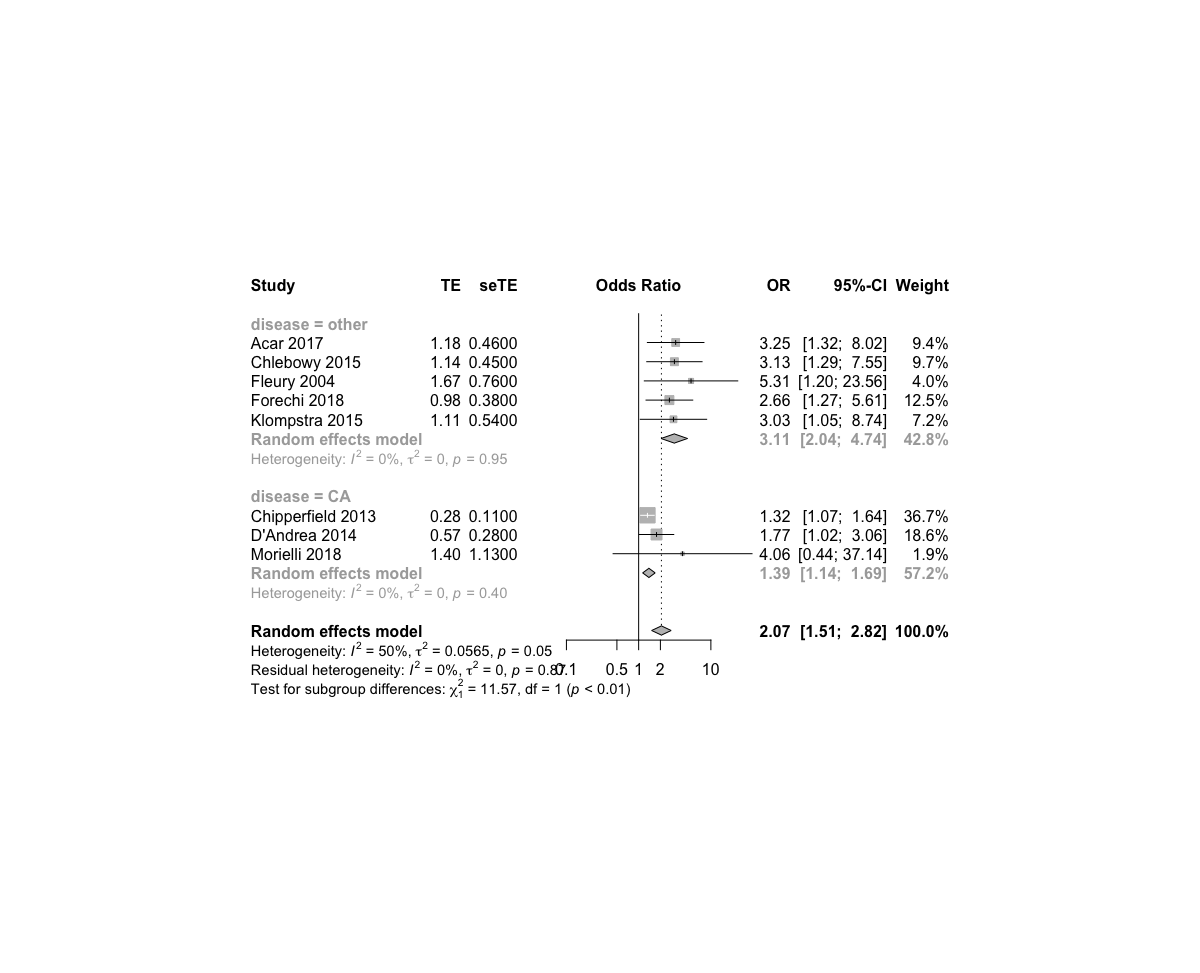
**

**Subgroup on design**

**
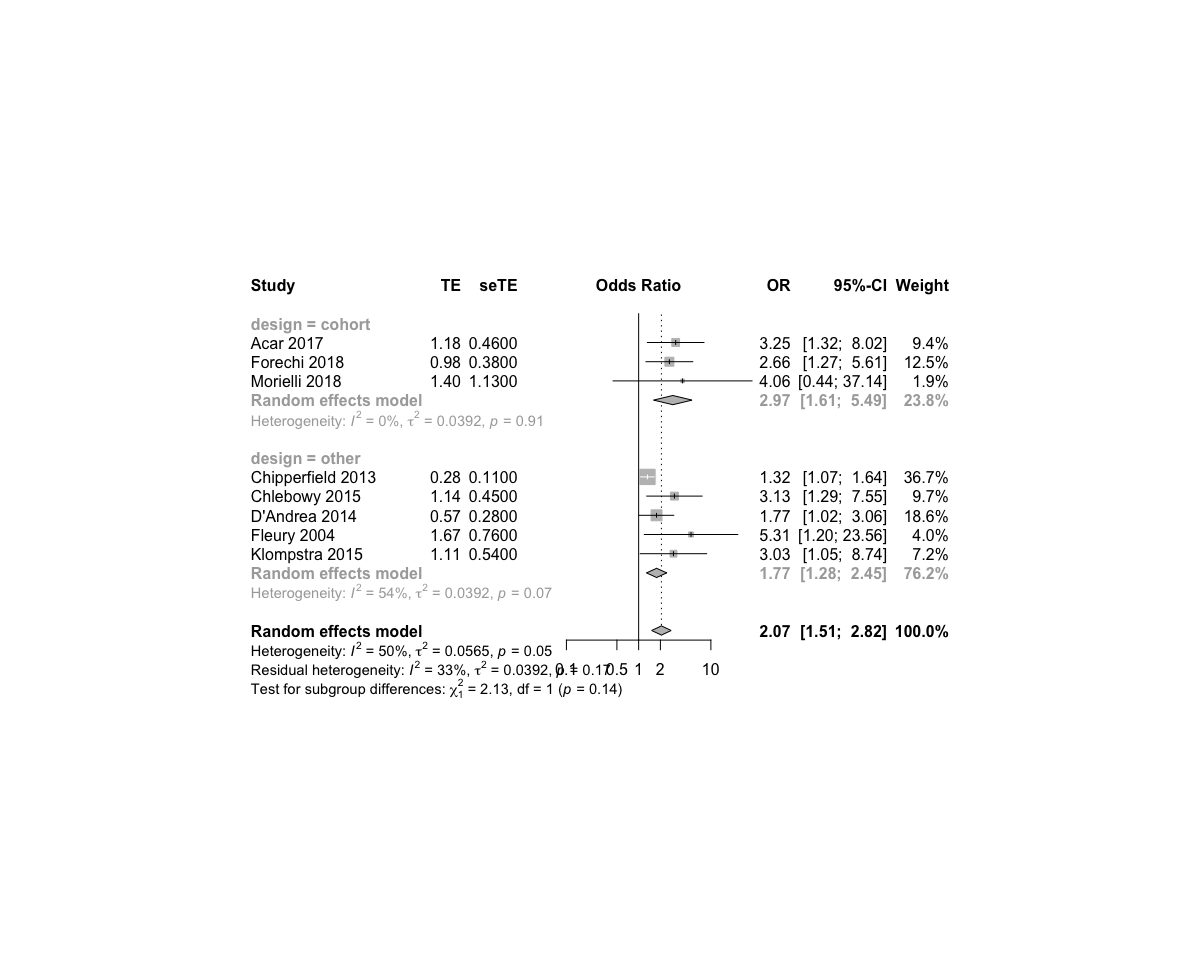
**
